# Ion channel noise shapes the electrical activity of endocrine cells

**DOI:** 10.1371/journal.pcbi.1007769

**Published:** 2020-04-06

**Authors:** David M. Richards, Jamie J. Walker, Joel Tabak

**Affiliations:** 1 Living Systems Institute, University of Exeter, Exeter, United Kingdom; 2 College of Engineering, Mathematics and Physical Sciences, University of Exeter, Exeter, United Kingdom; 3 Bristol Medical School, Translational Health Sciences, University of Bristol, Bristol, United Kingdom; 4 University of Exeter Medical School, University of Exeter, Exeter, United Kingdom; University of Pittsburgh, UNITED STATES

## Abstract

Endocrine cells in the pituitary gland typically display either spiking or bursting electrical activity, which is related to the level of hormone secretion. Recent work, which combines mathematical modelling with dynamic clamp experiments, suggests the difference is due to the presence or absence of a few large-conductance potassium channels. Since endocrine cells only contain a handful of these channels, it is likely that stochastic effects play an important role in the pattern of electrical activity. Here, for the first time, we explicitly determine the effect of such noise by studying a mathematical model that includes the realistic noisy opening and closing of ion channels. This allows us to investigate how noise affects the electrical activity, examine the origin of spiking and bursting, and determine which channel types are responsible for the greatest noise. Further, for the first time, we address the role of cell size in endocrine cell electrical activity, finding that larger cells typically display more bursting, while the smallest cells almost always only exhibit spiking behaviour.

## Author summary

The pituitary gland, situated just below the brain, is the body’s master hormone gland. Hormones produced by the pituitary control many essential functions, including growth, reproduction, and our response to emotional and physical stress. The cells that produce these hormones generate electrical activity, exactly like neurons, and this electrical activity controls the amount of hormone that is released. Here, we use mathematics and computing to help understand the electrical activity of these cells. This allows us to perform manipulations that we cannot do experimentally. In particular, we analyse a type of mathematical model that, for the first time, takes into account the role that is played by random processes within pituitary cells. These random processes are particularly important for these types of cell. Using this approach, we determine what causes the different types of electrical activity seen in pituitary cells. A particularly exciting aspect of this work is that it allows us, for the first time, to find out how the electrical activity of big cells is different to that for small cells. Long term, the aim of this work is to understand better how drugs affect hormone production and so suggest ways to reduce their side effects.

## Introduction

The crucial role of noise in many biological systems has only recently started to be fully appreciated [1]. Although noise is often averaged out at the macroscopic level, stochastic effects can become important at smaller scales or in cases where the component of interest is only present in a handful of copies. For example, noisy transcription and translation lead to noisy gene expression levels, which is often buffered by intricate regulatory networks [2]. Similarly, during development, maximising positional precision is likely to have exerted an evolutionary pressure on the shape of morphogen profiles [3]. In addition, noise can even be beneficial to biological systems, leading, for example, to quicker evolution in changing environments and improved signal detection [4, 5].

For electrically excitable cells, such as neurons, a major source of noise comes from stochastic ion channel kinetics [6]. Here, we study the effect of realistic ion channel noise in endocrine cells within the anterior pituitary. For these cells, the rate of hormone release is influenced by the pattern of membrane electrical activity, which in turn is controlled by the stochastic opening and closing of membrane ion channels. For example, gonadotroph cells produce spontaneous sharp action potentials (spikes) that cause little hormone secretion, whereas somatotrophs and lactotrophs exhibit spontaneous bursts in electrical activity that are sufficiently prolonged to elevate the intracellular calcium concentration and stimulate substantial hormone secretion [7].

Large-conductance potassium (BK) channels have recently been identified as the primary factor responsible for this difference in electrical activity between cell types. BK channels are expressed on somatotrophs and lactotrophs but not on gonadotrophs [7–9]. These channels are voltage- and calcium-gated and permit a rapid outward current that often shortens spike duration in many excitable cell types [10–15]. However, in some pituitary cell types, these channels paradoxically cause bursting activity [9]. We and others have shown, both experimentally with dynamic clamp and from mathematical modelling, that gradually increasing the total BK channel conductance in such cells can cause a transition from spiking to bursting [16, 17].

Due to the nature of BK channels, the role of noise in these cells is likely to be particularly significant for two reasons. First, the conductance of a single BK channel is around 100 pS, ten times greater than for other relevant channels. This means that the stochastic opening or closing of even a single BK channel will have a substantial effect on the potassium current. Second, since the total BK conductance can be as low as 0.5 nS, there could be as few as five active BK channels per cell. This should be compared to the other channels of interest, which are typically present at 200 or more per cell. The coefficient of variation (*i.e*. the relative standard deviation) for the number of open channels (for a total of N channels) behaves like $1/\sqrt{N}$, meaning that considerable fluctuations are expected for BK channels. Thus we expect noise in a system with BK channels to play an important role in determining the electrical activity and the nature of spiking and bursting.

Previous mathematical modelling has been remarkably successful in capturing many features of the observed electrical activity in pituitary cells. In particular, a number of recent models have included BK channels in the traditional Hodgkin-Huxley framework [9, 18, 19], studying how they affect electrical activity. These models led to the model by Tabak et al., which includes calcium channels, three types of potassium channels (including BK channels), and a varying intracellular calcium concentration [16]. This model explicitly shows how BK channels promote bursting behaviour: their relatively fast activation reduces depolarisation of the membrane potential, which limits activation of the other repolarising potassium channels, causing the potential to oscillate around a depolarised level, so leading to bursts. Further, as confirmed experimentally, this model demonstrates that gradually increasing the total BK conductance leads to greater and greater levels of bursting.

Although the role of channel noise in neurons [6, 20, 21] and pancreatic *β*-cells [22–25] has been studied, there is next to no work examining realistic channel noise in endocrine cells. With the exception of an exocytosis model (that considers only calcium channels rather than the membrane potential) [26], all previous models of endocrine cells have either been entirely deterministic or only included noise in an *ad hoc* manner via a normally-distributed fixed noise current. Such modelling approaches may be appropriate in cells with a high number of ion channels, but are unlikely to be sufficient in the present case. In particular, these models ignore the fact that noise in this system originates from the stochastic opening and closing of ion channels, and so completely miss how noise in different channels combines to give the overall stochastic behaviour. This makes it impossible to determine how noise affects the pattern of electrical activity and how this depends on parameters such as cell size and channel number.

Here, we address this by considering a realistic stochastic model of endocrine cells in the anterior pituitary. Rather than an overall noise current, we keep track of the state of every individual ion channel, each of which, at any given time, may be open or closed. Noise is added in a realistic manner by allowing each channel to independently open or close, with a transition probability related to the membrane potential or intracellular calcium concentration. Using this, we investigate the role of channel noise in pituitary cells, determine the relative importance of noise in different channels (particularly BK channels), and explore the effect of varying the total BK conductance. For the first time, as far as we are aware, we are also able to determine the effect of cell size on spiking and bursting behaviour, predicting that bursting is much more common in larger cells. Finally, we show that the effects of BK channel noise on bursting are robust to heterogeneous channel expression within a population of cells.

## Results

### Review of the deterministic model

We base our pituitary cell model on the model by Tabak et al. [16], a type of Hodgkin-Huxley model [27], where the membrane potential *V* is controlled by five currents: an L-type Ca^2+^ current (*I*_Ca_), three K^+^ currents (*I*_K_, *I*_SK_ and *I*_BK_) and a leak current (*I*_leak_). Then *V* is described by

$$\begin{array}{c} C\frac{dV}{dt}=-\left(I_{\text{Ca}}+I_{\text{K}}+I_{\text{SK}}+I_{\text{BK}}+I_{\text{leak}}\right), \end{array}$$ (1)

where *C* is the membrane capacitance. Note that, since we implement noise by directly considering the opening and closing of individual channels, we have no need for an explicit noise current. The Ca, K, and BK channels are assumed to be voltage-gated, whereas SK is Ca^2+^-gated. Although BK channels are known to be also Ca^2+^-gated, BK channels are usually adjacent to Ca^2+^ channels and so controlled by calcium microdomains [28] meaning that, since the Ca^2+^ concentration at BK channels then attains equilibrium in microseconds, BK channel activation can effectively be modelled as just voltage-gated [29, 30]. The difference between the K and BK channels lies in the single channel conductance (*g*_1,BK_ is twenty times larger than *g*_1,K_), the number of channels per cell (with hundreds of K channels and only ten or fewer BK channels), and the time constant (*τ*_*n*_ is six times longer than *τ*_BK_).

Activation of the Ca, K, SK, and BK channels are described by gating variables *m*, *n*, *s* and *f* respectively, each of which should be thought of as the probability that a given channel is open. These types of model often reduce the number of variables by replacing one or more gating variables by steady-state activation functions. However, since we wish to model realistic noise in all channels, we cannot do this. The Ca^2+^ and K^+^ currents are then given by

$$\begin{array}{c} \begin{array}{cc} I_{\text{Ca}}=g_{\text{Ca}}m\left(V-V_{\text{Ca}}\right), & I_{\text{K}}=g_{\text{K}}n\left(V-V_{\text{K}}\right),\\ I_{\text{SK}}=g_{\text{SK}}s\left(V-V_{\text{K}}\right), & I_{\text{BK}}=g_{\text{BK}}f\left(V-V_{\text{K}}\right), \end{array} \end{array}$$ (2)

and the leak current by *I*_leak_ = *g*_*l*_(*V* − *V*_*l*_). Here *V*_Y_ is the reversal potential for ion Y and *g*_X_ the maximal conductance for channel X. Here and in the following we will use to X to refer to an unspecified channel type (either Ca, K, SK or BK). The conductances are related to the single channel conductances via *g*_X_ = *g*_1,X_ *N*_X_, where *N*_X_ is the number of a given channel type.

The dynamics of the gating variables are governed by

$$\begin{array}{c} \begin{array}{cc} \tau_{m}\frac{dm}{dt}=m_{\infty }\left(V\right)-m, & \tau_{n}\frac{dn}{dt}=n_{\infty }\left(V\right)-n,\\ \tau_{s}\frac{ds}{dt}=s_{\infty }\left(\left[\text{Ca}\right]\right)-s, & \tau_{\text{BK}}\frac{df}{dt}=f_{\infty }\left(V\right)-f, \end{array} \end{array}$$ (3)

where *τ*_X_ are the time constants (assumed to be voltage-independent). The steady-state activation functions are given by

$$\begin{array}{c} \begin{array}{cc} m_{\infty }\left(V\right) & =[1+\text{exp}\;(\left(v_{m}-V\right)/s_{m}){]}^{-1},\\ n_{\infty }\left(V\right) & =[1+\text{exp}\;(\left(v_{n}-V\right)/s_{n}){]}^{-1},\\ f_{\infty }\left(V\right) & =[1+\text{exp}\;(\left(v_{f}-V\right)/s_{f}){]}^{-1},\\ s_{\infty }\left(\left[\text{Ca}\right]\right) & ={\left[\text{Ca}\right]}^{2}/({\left[\text{Ca}\right]}^{2}+k_{s}^{2}), \end{array} \end{array}$$ (4)

with *k*_*s*_, *v*_X_ and *s*_X_ the calcium-midpoint, voltage-midpoint and slope parameters respectively. The dependence of *s*_∞_ on [Ca] is as in previous models of both endocrine and pancreatic *β*-cells [18, 31].

The final model variable, the intracellular free Ca^2+^ ion concentration [Ca], evolves according to

$$\begin{array}{c} \frac{d\left[\text{Ca}\right]}{dt}=-f_{c}\left(\alpha I_{\text{Ca}}+k_{c}\left[\text{Ca}\right]\right), \end{array}$$ (5)

where the first term describes flux through Ca^2+^ channels and the second term captures calcium extrusion. Here *α* is the conversion factor from charge to concentration, *k*_*c*_ is the extrusion rate, and *f*_*c*_ is the fraction of intracellular calcium present as free Ca^2+^ ions. Note that *f*_*c*_ should not be confused with the BK gating variable *f*. In total, this leads to a six variable model: the membrane potential (*V*), the four gating variables (*m*, *n*, *s*, *f*), and the calcium concentration ([Ca]). For parameter values, initial conditions and how we solve this system numerically, see the Methods and S1 Text.

### The stochastic model: Adding ion channel noise

To understand the role of channel noise in pituitary cells, we determine the gating variables via a stochastic process. For each channel type this involves keeping track of the number of open channels. For concreteness, for the remainder of this section, consider just BK channels (described by *f*); the treatment of the other channel types is similar. The equation for *f* in Eq (3) can be rewritten

$$\begin{array}{c} \frac{df}{dt}=\beta \left(V\right)\left(1-f\right)-\gamma \left(V\right)f, \end{array}$$ (6)

where *β*(*V*) = *f*_∞_(*V*)/*τ*_BK_ and *γ*(*V*) = (1 − *f*_∞_(*V*))/*τ*_BK_. In this form, it is clear that this can be reinterpreted as a master equation with *f* the probability *P*_op_ that a given single BK channel is open and (1 − *f*) the probability *P*_cl_ it is closed:

$$\begin{array}{c} \frac{dP_{\text{op}}}{dt}=\beta \left(V\right)P_{\text{cl}}-\gamma \left(V\right)P_{\text{op}}. \end{array}$$ (7)

Seen as a two-state Markov process (with non-constant transition rates), we then have, for each channel,

$$\text{Channel}\;\text{closed}\overset{\beta \left(V\right)}{\underset{\gamma \left(V\right)}{⇄}}\text{Channel}\;\text{open}.$$ (8)

At any given time, *N*_op_ channels are open and *N*_cl_ are closed, with *N*_BK_ = *N*_op_ + *N*_cl_ the total (fixed) number of channels and the gating variable *f* given by *f* = *N*_op_/*N*_BK_. In a short time step Δ*t*, *β*(*V*)Δ*t* is approximately the probability that a single closed channel opens and *γ*(*V*)Δ*t* the probability that a single open channel closes. This assumes that the average number of channels opening/closing in Δ*t* is small. Then the probability that a single channel both opens and recloses (or vice versa) within a single time step can be ignored. To ensure this is the case, a suitably small Δ*t* is required. More exact simulation methods are then not required [32]. Assuming that channels open and close independently of each other, the change in the number of open channels (and hence the value of *f*) can then be simulated by drawing from a binomial distribution. See S1 Text for details.

The number of each channel type changes from cell to cell. We assume that, for each channel type, there is some approximately constant area density so that the channel number scales with the square of the cell size. (In the following we always take cell size to refer to the diameter.) Based on measured single and total conductances, we estimate that in a typical cell of diameter 10 *μ*m with *g*_BK_ = 0.5 nS there are about 5 BK channels, 200 SK channels, 640 K channels and 200 Ca channels (see Methods for details). We have checked that our conclusions are not altered if these values are different by up to a factor of two (see S1 Text).

### Channel noise smooths the spiking-bursting transition

**Fig 1:**
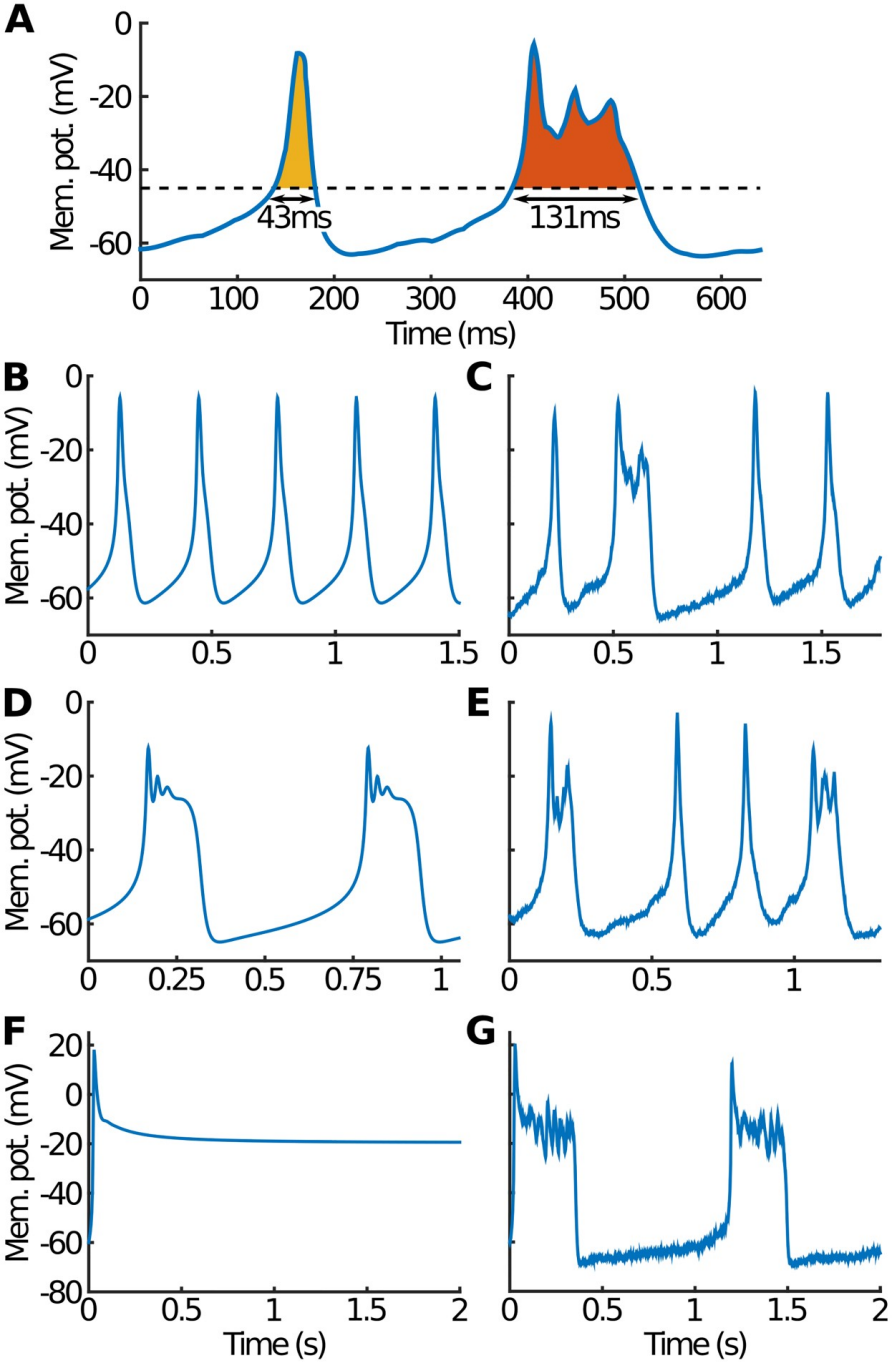
Spikes, bursts and the effect of noise. (A) The duration of an event is determined by the time that *V* spends above some threshold (chosen throughout as −45 mV). Events are classified as spikes if the event duration is less than 100 ms and there are no oscillations in *V* (*e.g*. first, yellow event). Events of longer duration or events with oscillations are identified as bursts (*e.g*. second, orange event). (B) The deterministic model with *g*_BK_ = 0.5 nS leads to pure periodic spiking. (C) The stochastic model with the same parameters shows that noise can convert some spikes into bursts. (D) Increasing *g*_BK_ to 1 nS leads to pure bursting in the deterministic model. (E) The equivalent stochastic model shows a mixture of spikes and bursts, with noise responsible for converting some bursts into spikes. (F) The deterministic model with *g*_Ca_ doubled to 4 nS simply sits at a depolarised steady state (*g*_BK_ = 0.5 nS). (G) However, the stochastic model with the same parameters shows bursts as noise continually drives the system away from the steady state.

We are interested in excitable behaviour where the membrane potential increases significantly from its resting value, enters a depolarised state and later returns to the resting state. We call such behaviour an event and define its duration using a threshold for *V* (Fig 1A). Events are caused by interplay between the calcium and potassium currents. At the start of an event, Ca^2+^ ions flow into the cell and so increase *V*. Later, the slower potassium currents respond by flowing out of the cell, decreasing *V* and so terminating the event. Two broad categories of event are observed: spikes, where the potential returns quickly to its resting value without any oscillations, and bursts, where significant time is spent in the depolarised state, often with oscillations around some depolarised value. For concreteness we here define spikes as events with duration less than 100 ms and no oscillations. Events of either longer duration or with oscillations are classified as bursts. Other definitions are possible but are unlikely to affect our conclusions.

The deterministic model has both periodic spiking and bursting solutions [16]. For example, consider varying the total BK conductance *g*_BK_ which, for fixed single channel conductance *g*_1,BK_, is equivalent to varying the number of BK channels *N*_BK_. At low *g*_BK_ (only a few BK channels) we find purely spiking behaviour just as in the classic Hodgkin-Huxley model (Fig 1B). However, at large *g*_BK_ (many BK channels) all spikes disappear giving rise to pure bursting (Fig 1D). This is caused by the relatively fast response of BK channels (*τ*_BK_ < *τ*_*n*_), which limits the initial membrane depolarisation. As a consequence, K channel activation is delayed, leading to longer events and oscillations in *V* around some depolarised value.

Turning on noise can dramatically change this behaviour. Although a simple implementation of noise has been studied before [16], this is the first time, as far as we are aware, that realistic channel noise with realistic numbers of channels has been considered in endocrine cells. For example, some of the spikes seen when *g*_BK_ is small can be converted into bursts (Fig 1C). Similarly, at large *g*_BK_, spikes can be seen in addition to bursts (Fig 1E). Further, even cases where deterministically there are no events at all (Fig 1F) can acquire events when noise is turned on (Fig 1G).

**Fig 2:**
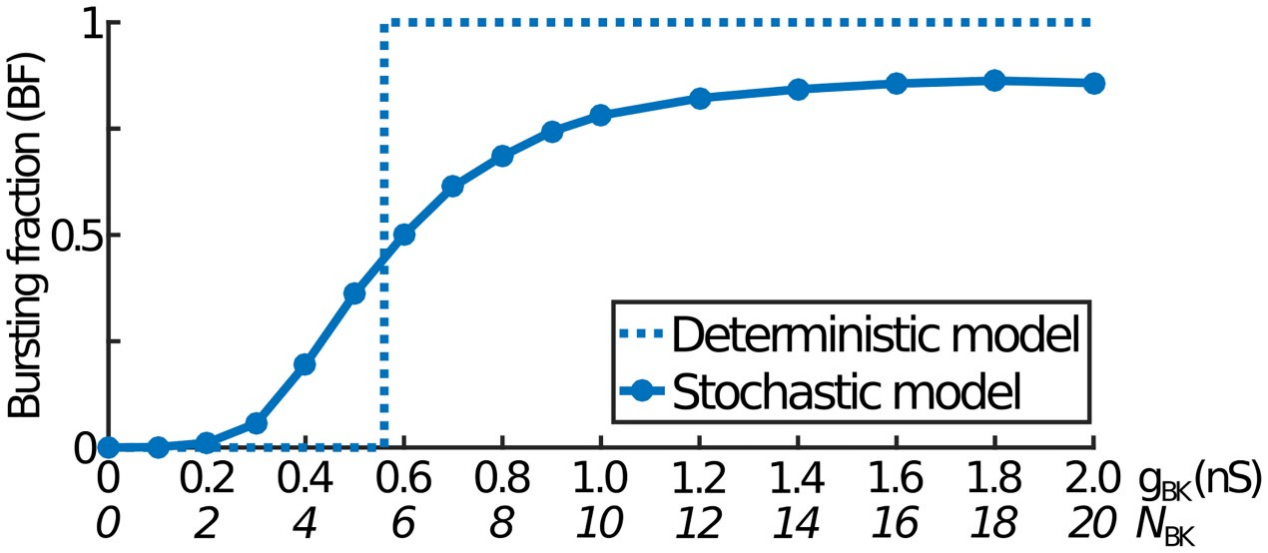
The bursting fraction against total BK conductance. In the deterministic model (dashed curve), the bursting fraction (BF) shows a sharp jump from pure spiking (BF = 0) to pure bursting (BF = 1) at a critical value of *g*_BK_. However, with channel noise (solid curve), this step function is smoothed out and no longer reaches pure bursting even for large *g*_BK_. Here, *g*_1,BK_ has been fixed throughout so that *g*_BK_ is proportional to *N*_BK_. Note that *N*_BK_ values only have a physical meaning in the stochastic model.

Solutions with both spikes and bursts lead to the concept of the bursting fraction (BF), defined as the fraction of events over a long time period that are bursts. Much of this paper will be concerned with how BF depends on model parameters and cell size. Almost all deterministic solutions that have events correspond to either pure spiking (BF = 0) or pure bursting (BF = 1) so that, as the BK conductance *g*_BK_ gradually increases, there is a critical point where BF suddenly switches from 0 to 1 (Fig 2). The inclusion of noise smooths out this behaviour and leads to two important differences. Firstly, bursts start at a lower value of *g*_BK_ due to noise converting some spikes into bursts. Secondly, BF = 1 is never reached, even at very high *g*_BK_: noise always converts some bursts to spikes. (This is in contrast to previous work where noise was treated less realistically, resulting in BF tending to 1 at large *g*_BK_ [16]). At first glance this seems counterintuitive: large *g*_BK_ corresponds to large numbers of BK channels and so should tend to the deterministic behaviour (BF = 1). However, although increasing *g*_BK_ increases *N*_BK_, the single channel conductance *g*_1,BK_ is unchanged. Thus, the change in current due to one BK channel opening or closing is always *g*_1,BK_(*V* − *V*_K_), independent of *N*_BK_, so that the proportion of bursts converted into spikes is similar for all large values of *N*_BK_.

### The number of channels and the BK time constant

Here we first consider the effect of the total number of channels on the bursting fraction. To do this, we scale the numbers of all four channel types by a factor *σ* so that {*N*_Ca_, *N*_K_, *N*_SK_, *N*_BK_} → {*σN*_Ca_, *σN*_K_, *σN*_SK_, *σN*_BK_} whilst keeping the total conductances fixed and ensuring that *σN*_X_ is an integer. This is equivalent to scaling all four single channel conductances by 1/*σ*. Scaling of single channel conductances whilst keeping the total conductances fixed is unlikely to be biological but shows the effect of the noise level on electrical activity. As *σ* → ∞ we expect to recover the deterministic model.

**Fig 3:**
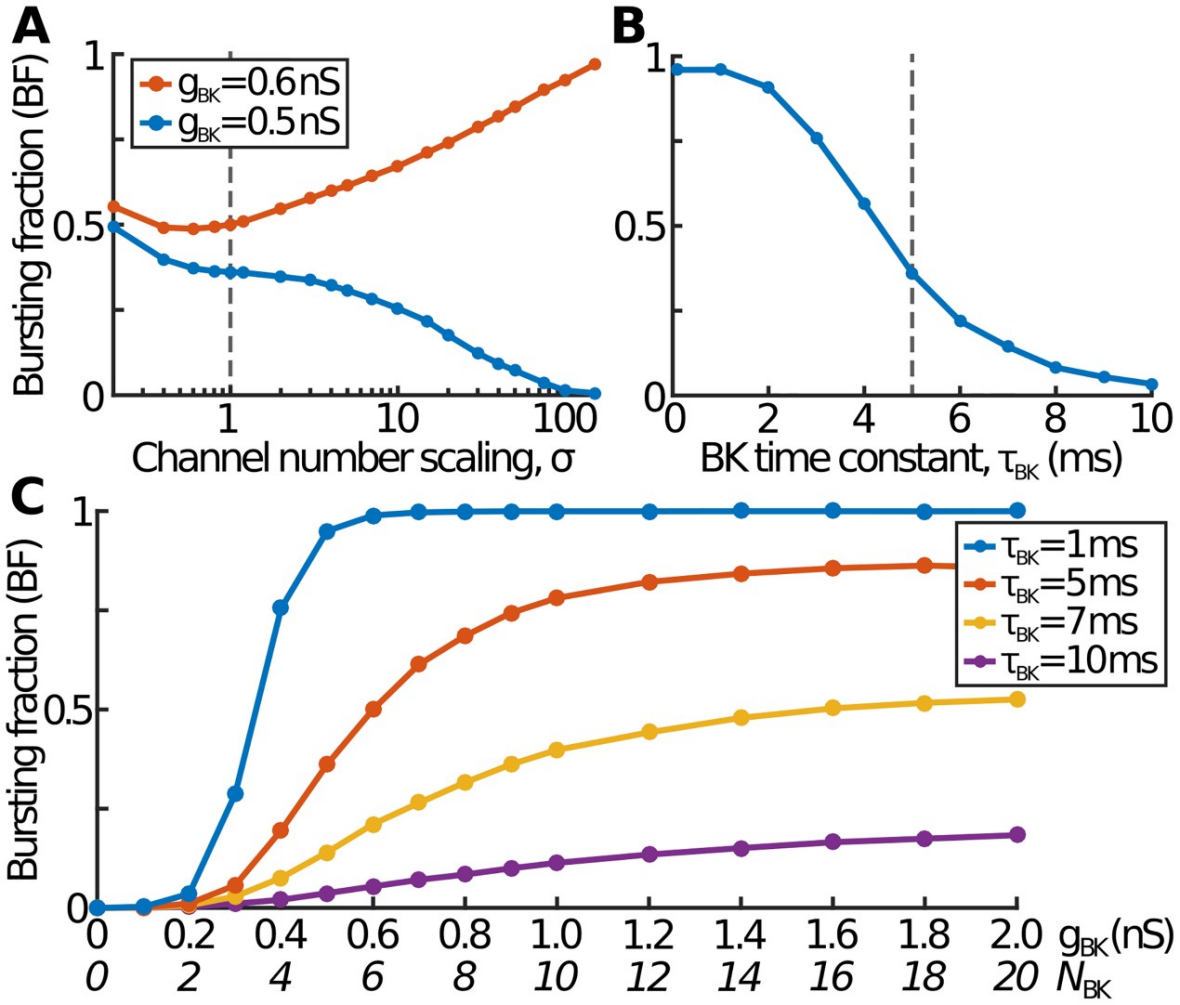
The effect on bursting of the total number of channels and the BK time constant. (A) For *g*_BK_ = 0.5 nS, corresponding to pure spiking in the deterministic model, BF gradually decreases as the total number of channels increases. However, for *g*_BK_ = 0.6 nS, corresponding to pure bursting deterministically, increasing *σ* leads to an initially drop in BF before an increase towards BF = 1. (B) Higher values of *τ*_BK_ lead to less bursting as BK channels start to act more like K channels. Conversely smaller *τ*_BK_, *i.e*. even faster acting BK channels, lead to more bursting. (C) The overall sigmoidal BF-against-*g*_BK_ behaviour seen in Fig 2 is the same for other values of *τ*_BK_. However, sufficiently large values of *τ*_BK_ only rarely lead to bursting even for very large total BK conductances.

First, consider the case when *g*_BK_ = 0.5 nS, which corresponds to pure spiking in the deterministic model. For the smallest values of *σ*, when there are only one or two BK channels, almost exactly half of events are spikes and half are bursts (Fig 3A). As *σ* increases, the bursting fraction gradually decreases to its deterministic value of BF = 0. Next, consider larger values of *g*_BK_, which lead to pure bursting in the deterministic case. Again, for small *σ*, about half of events are spikes/bursts (Fig 3A). As *σ* increases, BF increases towards 1 as expected, but not before first initially dipping slightly. This means that, for deterministically-bursting values of *g*_BK_, the least bursting corresponds to an intermediate number of channels, which in turn suggests that one way of controlling the bursting fraction involves careful choice of both the channel numbers and the single channel conductances.

In the deterministic model, and experimentally, we have shown that the BK channel needs to have fast opening and closing kinetics in order to have a burst-promoting effect [9, 16]. We now examine the effect of the BK time constant, *τ*_BK_ in our stochastic model. Larger *τ*_BK_ values mean that the BK channels start to behave more like the slower-acting K channels. Thus they are less likely to convert spikes to bursts, resulting in lower BF (Fig 3B). Conversely, smaller *τ*_BK_ correspond to very fast-acting BK channels and a tendency for almost all events to be bursts. This is in agreement with the deterministic model [16]. The effect on the sigmoidal BF-against-*g*_BK_ behaviour in Fig 2 is similar: small *τ*_BK_ result in effectively pure bursting even for modest values of *g*_BK_, whereas large *τ*_BK_ require larger *g*_BK_ before any noticeable bursting and, even for very large *g*_BK_, only occasionally generate bursts (Fig 3C).

### Individual channel types: BK versus other channels

The above results cover the realistic case of stochastic opening and closing in all four channel types that we consider here. However, with our model, we are able to determine the relative effect of noise in different channels. For example, we can examine whether noise in BK channels is more likely to cause bursting than noise in other channels. We do this by allowing some channel types to be described stochastically (as described above) and others deterministically (by considering the finite difference version of Eq (3)). In particular, we focus on comparing noise only in BK channels (with deterministic Ca, K and SK channels) with noise only in Ca, K and SK channels (with BK deterministic). We refer to the first case as “BK noise” and the second as “non-BK noise”.

**Fig 4:**
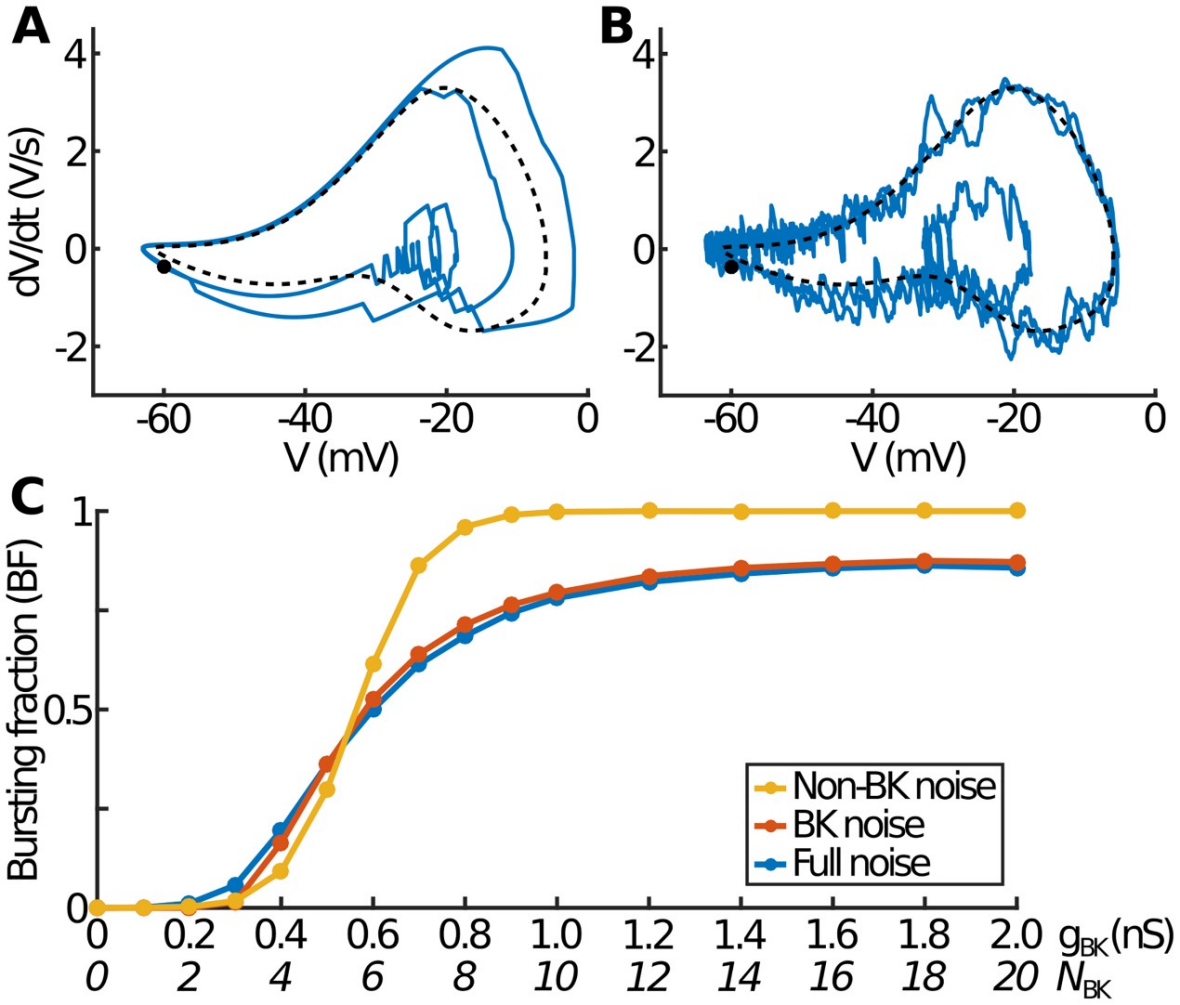
Relative noise contributions for BK versus non-BK channels. (A) Plot of *dV*/*dt* against *V* with *g*_BK_ = 0.5 nS and noise only in BK channels (Ca, K and SK behave deterministically) for a short 600 ms period during which two events occur (a spike followed by a burst). The dashed line shows the entirely deterministic solution (with no noise in any channels), which corresponds to continuous spiking. The black circle labels the initial starting configuration. (B) As in (A) but with noise only in the Ca, K and SK channels (BK acts deterministically). (C) The BF-against-*g*_BK_ behaviour for different sources of noise showing that, whereas non-BK noise slightly smooths out the deterministic step function, the majority of the difference between the deterministic and fully stochastic models arises from noise in the BK channels.

First, consider the plot of the time derivative of the membrane potential ($\frac{dV}{dt}$) against the potential itself (*V*). For the case of pure spiking in the deterministic model (*e.g*. with *g*_BK_ = 0.5 nS) the $\frac{dV}{dt}$-*V* trajectory is a single, non-intersecting loop (the dashed line in Fig 4A). This contrasts with the pure bursting case (*e.g*. with *g*_BK_ = 0.6 nS) where the trajectory self-intersects as *V* oscillates around a depolarised state. With the inclusion of stochastic channels, these trajectories become noisy. However, the precise nature of how noise affects the trajectories depends on exactly which channels are stochastic. For non-BK noise, the trajectory shows considerable fluctuation at all points during the cycle (Fig 4B). However, for BK noise, the trajectory is much smoother, with most of the fluctuation occurring while in the depolarised state (Fig 4A). This is related both to the fact that (*V* − *V*_K_) is small when in the resting state and to the fact that, during most of the cycle, when *V* is close to the resting potential, there are rarely any BK channels open; this, in turn, is because the steady state activation of BK channels (*f*_∞_ in Eq (4)) is very steep compared to that for other channels (since *s*_*f*_ is small compared to *s*_*m*_ and *s*_*n*_). Only once an event has started and *V* begins to rise, does the stochastic nature of BK channels become apparent and lead to noticeable differences from the deterministic model.

Now consider the maximum potential *V*_max_ during an event, which, due to noise, varies from event to event. For non-BK noise, *V*_max_ is similar for spikes and bursts. For example, for *g*_BK_ = 0.5 nS, the average of the *V*_max_ distribution is −5.6 mV for spikes and −5.9 mV for bursts, with a standard deviation of 1.1 mV in both cases. This should be compared to the deterministic result (corresponding to pure spiking), where *V*_max_ = −5.9 mV. The situation is different for BK noise, where the average *V*_max_ for bursts (−7.3 mV) is significantly lower than that for spikes (−4.8 mV). Again, the standard deviation (2.4 mV) is similar in each case. This difference between BK and non-BK noise also manifests itself in the BF-against-*V*_max_ histogram, which is approximately uniform for noise in non-BK channels and monotonically decreases towards 0 for BK noise. These results are similar for other values of *g*_BK_ and show that the way noise converts an event that would deterministically be a spike into a burst is different for BK and non-BK channels. For the case of BK noise, a burst is often caused by the sudden noisy opening of a BK channel as *V* is rising towards its maximum. This reduces *V*_max_, which in turn delays K channel activation and so prolongs the event. Conversely, for non-BK noise, bursts are typically due to the noisy opening or closing of a non-BK channel only once *V* has reached its maximum and has started to drop back towards its resting value.

The difference between BK and non-BK noise can also be seen in the distribution of event durations. In all cases this distribution ranges from approximately 50 ms for the shortest spikes to about 250 ms for the longest bursts. For concreteness, consider *g*_BK_ = 0.5 nS, which shows both spiking and bursting events. For the case of BK noise, the distribution of event durations is unimodal and fairly uniform. However, for non-BK noise this distribution is distinctly bimodal, with a clear separation between events that are spikes and events that are bursts.

Finally, we examine the BF-versus-*N*_BK_ curve. The effect of either BK or non-BK noise lies somewhere between the deterministic result (dashed line in Fig 2) and the full noise case (solid line in Fig 2), with both smoothing the deterministic step function to a greater or lesser extent (Fig 4C). However, non-BK noise only slightly differs from the deterministic case (around the spiking-bursting transition), with BF tending towards pure bursting for large *N*_BK_. In contrast, the case of BK noise looks almost identical to the full noise case. Thus, although noise in non-BK channels accounts for most of the fluctuation in the $\frac{dV}{dt}$-*V* trajectory (Fig 4B), it is BK channel stochasticity that determines the fraction of events that are bursts and typically causes events that would be spikes (or equivalently bursts) in the deterministic model to sometimes instead become bursts (or spikes). As expected, the fact that BK channels have a greater effect than other channels is due to their scarcity and relatively large single channel conductance.

### The role of cell size

Mathematical models of endocrine pituitary cells often neglect the role of cell size, even though there are substantial variations in the size of these cells, with cell diameters ranging from 7 to 15 *μ*m [33, 34]. Cell size varies not only between cell types but also within a given cell type and, for many organisms, depends on age, sex and phase within the reproductive cycle [35–37]. Further, pituitary adenomas and other pituitary tumours are often associated with changes in cell size [38].

We now explore the effect of cell size on electrical activity. We assume that cell size can be described by some effective radius *R*. It is not necessary that the cell is spherical, only that its membrane area and volume are proportional to *R*^2^ and *R*^3^ respectively. As our base case, we take a cell with diameter 2*R* = 10 *μ*m and consider scaling the radius by a factor λ so that *R* → λ*R*. Then the membrane area and cell volume scale by λ^2^ and λ^3^ respectively.

This scaling is likely to affect only some parameters. For example, we assume that the reversal potential and current gating parameters (voltage and calcium midpoints and slopes) are unaffected. However, other parameters, such as the conductances and membrane capacitance, will change. In particular, assuming a constant area density of membrane channels, we expect that both the numbers of channels *N*_X_ and therefore the conductances *g*_X_ scale with the membrane area. This also applies to the leak conductance. The membrane capacitance, *C*, which is the charge that the membrane stores per unit electric potential, also scales with membrane area.

Since the charge-to-molar conversion in the [Ca] equation, *α*, converts a current to an intracellular Ca^2+^ concentration, it follows that *α* scales inversely with the cell volume. In addition, assuming calcium extrusion occurs through the Na^+^/Ca^2+^ exchanger (NCX) and the plasma membrane calcium ATPase (PMCA), both of which operate through the membrane, we expect the Ca^2+^ extrusion rate *k*_*c*_ to scale like the membrane area over the cell volume. This is because the extruded calcium leaves through the membrane but affects the calcium concentration throughout the whole cell. (We have here assumed that the calcium dynamics occur throughout the whole cell volume. Although we believe this to be the most likely scenario, it is possible instead that the relevant changes in [Ca] are confined to a thin shell region adjacent to the membrane. This would lead to a different, although still nontrivial, dependence on cell size. See S1 Text for a full discussion of this.)

Thus, in summary, scaling *R* → λ*R* implies that

$$\begin{array}{c} \begin{array}{ccc} C\to \lambda^{2}C,\; & g_{\text{X}}\to \lambda^{2}g_{\text{X}},\; & g_{l}\to \lambda^{2}g_{l},\\ \alpha \to \alpha /\lambda^{3},\; & k_{c}\to k_{c}/\lambda ,\; & N_{\text{X}}\to \lambda^{2}N_{\text{X}}. \end{array} \end{array}$$ (9)

Since the membrane capacitance and conductances both scale with λ^2^, the equation for the membrane potential (Eq (1)) is unaffected by the *R* → λ*R* scaling. This is also the case for the *m*, *n*, *s* and *f* gating variables. However, the [Ca] equation (Eq (5)) acquires an overall 1/λ scaling on the right-hand side. It is worth noting that, if we only focus on the six model variables (*V*, *m*, *n*, *s*, *f*, [Ca]) and ignore other quantities such as the currents, this can equivalently be seen as a 1/λ scaling to the *f*_*c*_ parameter (the fraction of intracellular calcium present as free ions). Then the overall effect of scaling *R* → λ*R* is equivalent to simply scaling

$$\begin{array}{c} f_{c}\to f_{c}/\lambda ,\;N_{\text{X}}\to \lambda^{2}N_{\text{X}}. \end{array}$$ (10)

Of course, this does not mean that the intracellular ratio of free to bound calcium depends on the cell size, simply that this scaling of *f*_*c*_ gives an equivalent mathematical model.

### Cell size in the deterministic model

We now study the consequences of cell size on spiking and bursting behaviour. First, we investigate the deterministic model. Since this is equivalent to the limit *N*_X_ → ∞, the only effect of scaling *R* → λ*R* is to send *f*_*c*_ → *f*_*c*_/λ. This sharply contrasts with the classic deterministic Hodgkin-Huxley model, where the absence of an equation for [Ca] means there is no effect of scaling *R* and so identical dynamics for all cell sizes. It is precisely because this is not the case in the present model that makes the role of cell size, even in the deterministic version, an interesting topic.

For concreteness, consider the case where *g*_BK_ = 0.5 nS at λ = 1, which produces spiking in the deterministic model. First, we examine what happens as the cell size is increased (λ > 1), with a corresponding increase in *g*_BK_ (Eq (9)). This is equivalent to reducing *f*_*c*_ and so effectively results in slower calcium dynamics. As a consequence, once an event starts, the intracellular calcium concentration increases slower than for λ = 1, which causes *s*_∞_ (and so *s*) to also increase slower. In turn, this means that *I*_SK_ rises more slowly, so that *V* remains longer in the depolarised state and so leads to longer events. Modest increases in λ still correspond to spiking (although with longer event durations), but for sufficiently large λ there is a sudden transition to pure bursting. Further increasing λ results in longer and longer bursts. It is noteworthy that the mechanism by which spiking transitions to bursting when λ is increased is different to that when *g*_BK_ is increased by itself: the former is related to slower calcium dynamics, whereas the latter involves a larger BK current and so lower *V*_max_.

**Fig 5:**
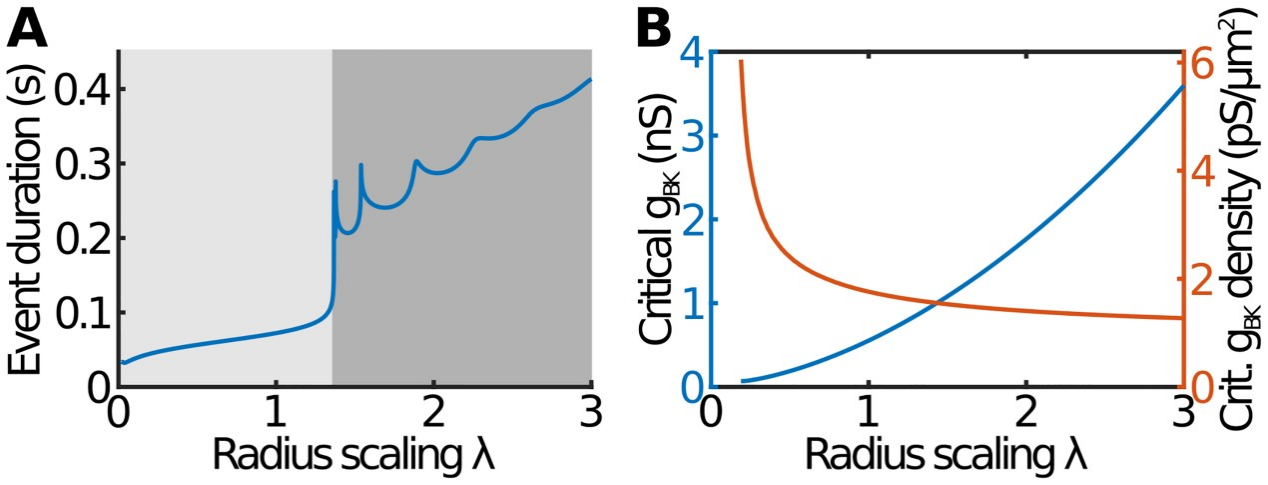
The effect of cell size in the deterministic model. (A) Event duration against cell size for the case where *g*_BK_ = 0.5 nS at λ = 1. The spiking behaviour switches sharply to bursting at around λ = 1.35. Light shading: pure spiking; dark shading: pure bursting. (B) The critical BK conductance for switching between spiking and bursting as a function of the cell size. The blue curve (left axis) shows the total BK conductance within the cell (*g*_BK_), whereas the red curve (right axis) shows the BK conductance area density (*g*_BK_/4*πR*^2^).

Now consider the case of smaller cells (λ < 1). This is equivalent to quicker calcium dynamics, which in turn leads to shorter events. This means that spiking still continues for smaller cells, although with shorter durations. Only for λ < 0.02 (which corresponds to unnaturally small, submicron-sized cells) does the spiking behaviour break down, with the cell instead entering a permanently depolarised state with *V* ≈ −45 mV. These results for both smaller and larger cells are summarised in Fig 5A, which shows that the transition to bursting is accompanied by a sharp increase in event duration. The non-monotonic behaviour within the bursting region is due to oscillations of *V* within the depolarised state: each maximum corresponds to the appearance of an extra oscillation.

Finally, we consider how, in the deterministic model, the transition between spiking and bursting depends on cell size. For a given λ, there is a critical conductance, $g_{BK}^{*}$, at which spiking behaviour sharply switches to bursting (see the dashed curve in Fig 2). Since the total BK conductance scales with cell size (Eq (9)), it makes more sense to consider the *g*_BK_ area density (*i.e*. the BK conductance due to a unit area of membrane), defined as *g*_BK_/4*πR*^2^. To calculate this, we identify λ = 1 with a diameter of 10 *μ*m, although our qualitative results are not dependent on this choice. In Fig 5B we plot how both the critical *g*_BK_ and the critical *g*_BK_ density depend on λ. As expected, larger cells (with more channels) require larger $g_{BK}^{*}$ to transition from spiking to bursting. However, interestingly, the critical *g*_BK_ density (*i.e*. $g_{BK}^{*}/4\pi R^{2}$) decreases with increasing cell size, suggesting that larger cells are more likely to exhibit bursting than smaller cells. This can again be understood from the effectively slower calcium dynamics in larger cells, which leads to longer events and means that the spiking-to-bursting transition occurs at lower *g*_BK_ densities.

### Cell size in the stochastic model

**Fig 6:**
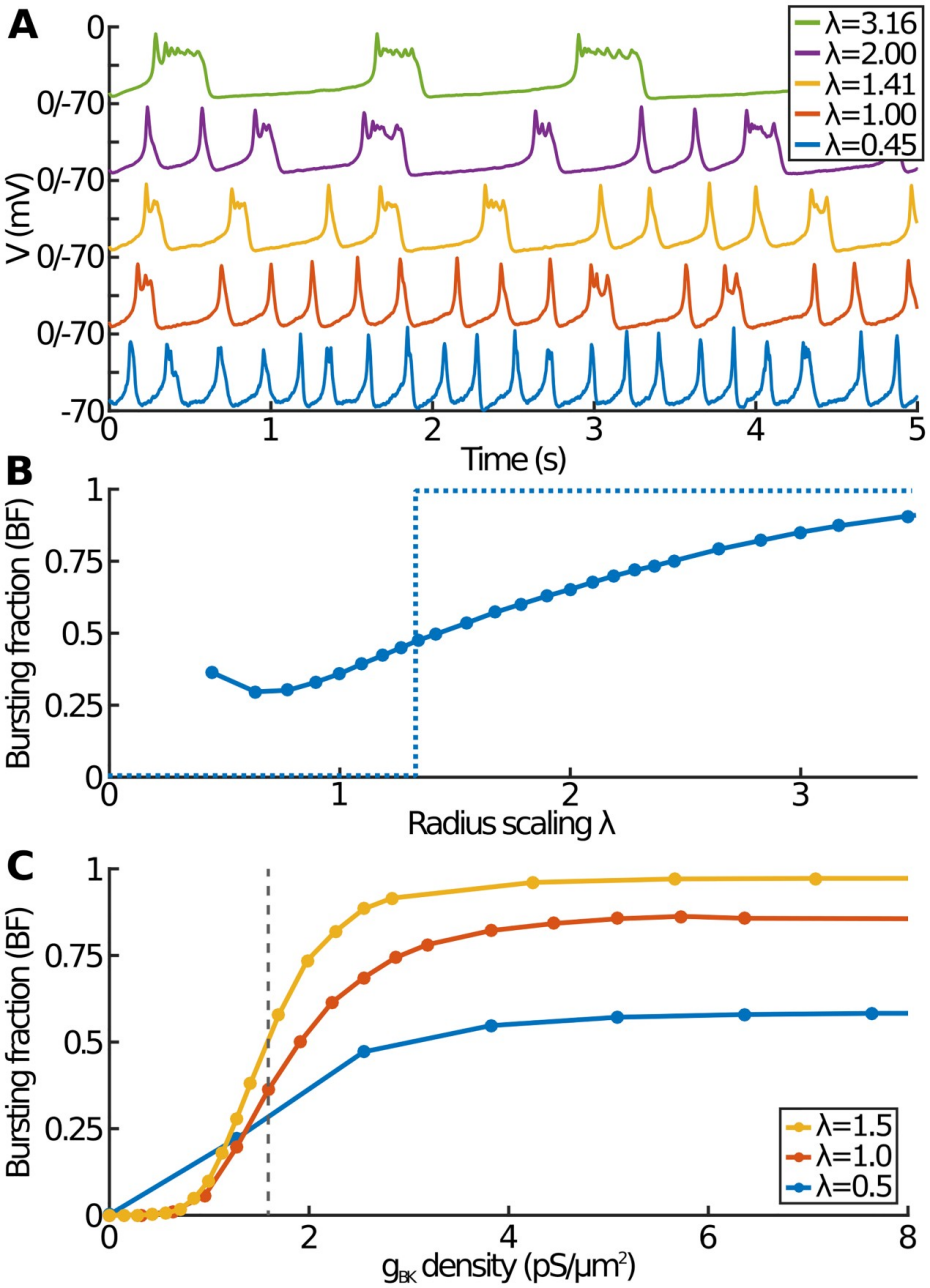
The effect of cell size in the stochastic model. (A) Examples of the membrane potential from simulations of five different cell sizes, showing mostly spiking for the smallest cells (λ = 0.45) and gradually becoming almost complete bursting for large cells (λ = 3.16). For each trace, the y-axis ranges from −70 to 0 mV. (B) The bursting fraction as a function of the cell size based on a 10 *μ*m cell (λ = 1) having 5 BK channels. The dashed line shows the equivalent plot for the deterministic model. (C) The bursting fraction as a function of the *g*_BK_ area density for three cell sizes. Panel (B) corresponds to BF measured along the dashed vertical line in Panel (C).

We now consider the effect of cell size in the more realistic stochastic model. Only values of λ that lead (without rounding) to an integer number of channels (for all channel types) are considered. In particular, since we take *N*_BK_ = 5 at λ = 1, we do not consider cells smaller than λ^2^ = 1/5; such cells would not even have one BK channel. The effect of changing λ now not only affects the rate of calcium dynamics, but also changes the number of channels (Eq (10)). In Fig 6A we plot example voltage traces for five different cell sizes. As usual, we set *g*_BK_ = 0.5 nS at λ = 1. As expected, there is less noise in the *V* time course for larger cells due to the greater number of channels. Further, larger cells produce more bursts and generally longer events.

To examine this further, we plot the bursting fraction against cell size (Fig 6B). It is worth noting that, based on a 10 *μm* cell for λ = 1, the extremes of this plot correspond to unrealistically small (<5 *μ*m) and large (>30 *μ*m) cells. As cell size increases, BF increases towards pure bursting. This is for exactly the same reasons as in the deterministic model: increasing λ corresponds to slower calcium dynamics, which in turn leads to a slower rise in the SK current, giving rise to longer events that are more likely to be bursts. Similarly, up to a point, smaller cells result in shorter events and so lower bursting fractions. However, interestingly, for the smallest cells, BF again starts to rise. This is because smaller cells have fewer channels, meaning that stochastic effects become more important. BF is then determined by competing effects between the calcium dynamics and the number of channels: for small cells, in addition to slow calcium dynamics, which tends to reduce event duration (and so reduce BF), there are also fewer channels (in particular BK channels), so that channel noise is more likely to convert would-be spikes into bursts (and so increase BF). This increase in bursting for the smallest cells is completely missed in the deterministic model and shows that the most spiking (smallest BF) is found in intermediate-sized cells.

We also plot BF against the *g*_BK_ density for various cell sizes (Fig 6C). Fig 6B can be thought of as Fig 6C along the dotted vertical line. In each case, we obtain the familiar smoothed Heaviside function with, except for the smallest *g*_BK_ densities, more bursting in larger cells. It is notable that the *g*_BK_ density at which bursts first appear (*i.e*. the point where BF first differs noticeably from 0) is approximately the same for all three cell sizes. In addition to our model predicting that large cells burst more, we also find that, in the smallest cells, at most approximately half the events can be bursts even for the highest *g*_BK_ densities, in stark contrast to the deterministic model.

### The effect of opening and closing individual channels

Ion channel noise is able to convert would-be spikes into bursts and vice versa. In this section we examine, both in the deterministic and stochastic models, how this is related to the opening and closing of individual ion channels. We determine at what point during an action potential noise is most effective in converting a would-be spike into a burst and vice versa. For concreteness we focus on the case *g*_BK_ = 0.5 nS, which corresponds to pure spiking in the deterministic model.

In particular, we study the effect of manually perturbing the system at a particular point during an event and in a particular channel. The perturbation involves either opening/closing a given number of channels or injecting a current for a short fixed period of time. The point to apply the perturbation is determined from a potential threshold V*, with the perturbation applied the first time during each event that the potential crosses V*. In turn V* can be mapped, using the deterministic spike action potential, onto a time before or after *V*_max_. For negative times (*i.e*. times whilst the deterministic potential is rising towards *V*_max_), the perturbation is applied the first time *V* rises above V*. Conversely, for positive times (*i.e*. times whilst the deterministic potential is dropping from *V*_max_), the perturbation is applied the first time (since *V*_max_) that the potential drops below V*. This ensures that there is at most one perturbation per event.

In the stochastic model, for a given channel type, the size of the fluctuations of the number of open channels depends on the total number of channels and so on the channel type. For example, consider the BK channels. At any given time, at least if we assume a quasi-steady state, we expect the number of open BK channels to be approximately binomially distributed with mean *f*_∞_*N*_BK_ and variance *f*_∞_(1 − *f*_∞_)*N*_BK_ (see S1 Text). Thus the maximum standard deviation (which occurs when *f*_∞_ = 0.5) is $\frac{1}{2}\sqrt{N_{\text{BK}}}$. This suggests that, to make the size of the perturbation comparable across different channel types, we should take the perturbation for channel X to be $\frac{1}{2}\sqrt{N_{\text{x}}}$. In particular, this means that we take a perturbation to consist of simultaneously opening or closing 7 Ca channels, 13 K channels, 7 SK channels or 1 BK channel.

**Fig 7:**
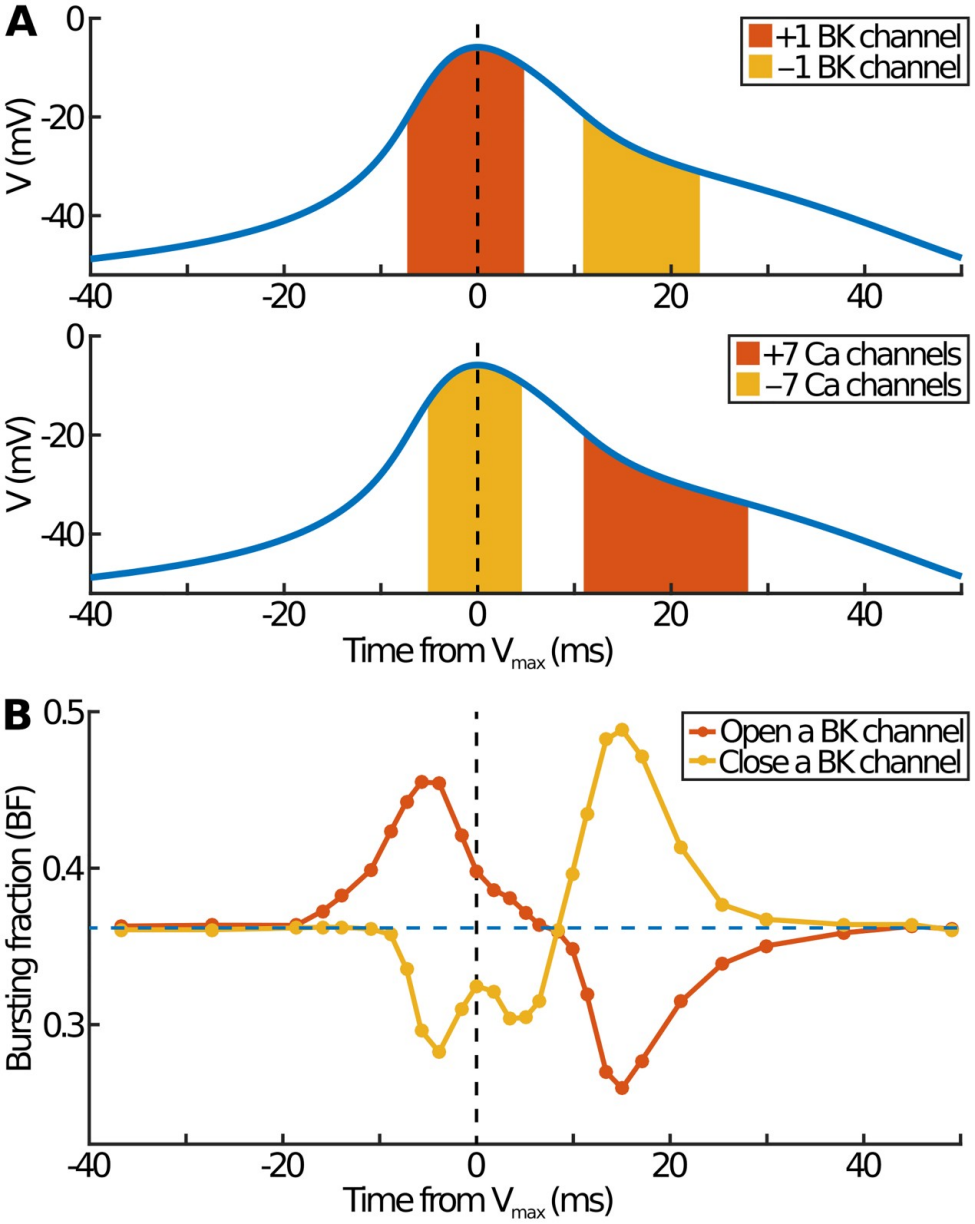
The effect of opening and closing individual channels. (A) Ranges over which opening or closing channels in the deterministic model causes spikes to become bursts. The blue curve shows the (non-perturbed) deterministic *V* profile, which corresponds to a spike. The horizontal axis shows the point during the action potential when the perturbation (here implemented as an extra current) is applied. Red/yellow regions: times from *V*_max_ when adding/removing a current converts spiking to bursting. Top/bottom: perturbation applied in BK/Ca channels. In each case the perturbation is applied for 5 ms. (B) Effect on BF in the stochastic model of opening/closing a single BK channel at different points during the action potential. The horizontal axis shows the point when the perturbation is applied: a time from *V*_max_ corresponds to a particular value of the potential in the deterministic model (blue curves in (A)); the perturbation is applied when the potential first rises above or drops below this value. Blue horizontal dashed line: value of BF with no perturbation.

Consider first the deterministic model. Based on Eq (2), we implement a perturbation in channel X by injecting a current *g*_X_(*q*/*N*_X_)(*V* − *V*_X_) for 5 ms. Here *q* is the change in the number of open channels: positive/negative *q* corresponds to opening/closing *q* channels. Whether the perturbation transforms spikes into bursts depends both on *q* and on the time the perturbation is applied (*i.e*. on V*). In Fig 7A we plot the regions where bursting occurs along with the deterministic spike potential.

For potassium channels (upper panel in Fig 7A), there is a region around *V*_max_ where transiently opening channels leads to bursting. This works in much the same way that BK channels cause bursting: the perturbation lowers *V*, which delays subsequent activation of the repolarising currents, and so extends the event duration. This effect only applies within 5 to 10 ms of *V*_max_: perturbations that are applied too early or too late make little difference to the event profile. Similarly there is a region after *V*_max_ (as *V* is dropping back towards the resting state) when closing potassium channels leads to bursts. In this case the applied current acts to increase *V* and so maintains the cell in the depolarised state for longer. The size of the regions that lead to bursting varies depending on the channel type. Opening or closing a single BK channel leads to wider regions than opening or closing seven SK channels, which in turn leads to wider regions than opening or closing 13 K channels. This difference boils down to the relative size of *g*_X_(*q*/*N*_X_), which is proportional to $g_{1,\text{x}}\sqrt{N_{\text{x}}}$.

For calcium channels (lower panel in Fig 7A), we instead find the opposite effect: manually opening Ca channels leads to bursting only for a time after *V*_max_, whereas closing Ca channels causes bursts when *V* is around its maximum. This corresponds to the fact that, for *V* near *V*_max_, *I*_Ca_ is negative (calcium ions flow into the cell) whereas *I*_K/SK/BK_ is positive (potassium ions flow out of the cell). Interestingly the range within which opening Ca channels after *V*_max_ causes bursting is significantly larger than that for closing BK channels.

We now consider perturbations in the full stochastic model and determine how these affect BF as a function of when they are applied. In particular, we plot BF against the time in the deterministic model when the potential first crosses V* (see Fig 7B for the BK case). Rather than using currents, perturbations are now implemented as the direct opening or closing of a given number of channels. When potassium channels are opened/closed before or around *V*_max_, BF increases/decreases, whereas when they are opened/closed after *V*_max_, BF is decreased/increased. Again, the opposite is seen for calcium channels. The point at which perturbations make no change to BF depends on channel type and does not correspond to *V*_max_. For example, for BK channels, a perturbation applied about 8 ms after *V*_max_ does not affect BF. This does not mean that these perturbations do not cause spikes to become bursts and vice versa, simply that the number of spikes converted to bursts is offset by the number of bursts converted to spikes.

Not surprisingly, the size of perturbation required for an observable change to BF depends on the channel type: perturbations of only one K or BK channel notably alter BF, whereas more than 40 Ca or SK channels must be simultaneously opened or closed for any noticeable effect. This is related to the very short time constants for Ca and SK channels (*τ*_*m*_ and *τ*_*s*_), which mean that modest perturbations in these channels are quickly rectified. This is not the case for the K and BK channels, where *τ*_*n*_ and *τ*_BK_ are much longer.

The size of the shift in BF often depends on whether the perturbation is before or after *V*_max_. For example, for K and Ca channels, perturbations have the largest effect when applied after *V*_max_, with a much smaller effect for perturbations before *V*_max_. This is not the case for BK channels with approximately equal shift in BF (see Fig 7B). The situation for SK channels is difficult to determine since even simultaneously opening or closing 100 channels has little effect on BF. These results match well with those in the “Individual channel types: BK versus other channels” section where we considered the effect due to noise in only certain channel types at a time.

Finally, it is worth noting the small local maximum when the perturbation involves closing a BK channel at *V*_max_ (yellow curve in Fig 7B). The equivalent is not seen when a BK channel is opened. This is because, for V* near *V*_max_, not all events reach a high enough *V* for the perturbation to be applied. In particular, when a BK channel is closed near *V*_max_ the effect is to convert some bursts into spikes and so reduce BF. However, because bursts tend to have lower *V*_max_ than spikes, some bursts avoid the perturbation and so remain bursts, meaning that BF is not reduced as much as might initially be thought. The opposite effect due to opening a BK channel near *V*_max_ is not seen since would-be spikes are more likely to cross the relatively high threshold value that triggers the perturbation.

In summary, we have been able to shed some light on the role channel noise plays in determining whether an event is a spike or a burst, and how this depends on channel type and fluctuation size. As expected, BK channels have the most significant effect, with the opening or closing of even a single channel often substantially affecting BF. In particular, the greatest effect occurs for BK perturbations either about 4 ms before *V*_max_ or 15 ms after. By contrast ten or more K channels must simultaneously be opened to observe a similar effect, and even then only for perturbations applied after *V*_max_. Finally, the short time constants for the Ca and SK channels mean that noise in these channels typically has little effect on whether an event is a spike or a burst.

### Robustness of results to parameter variations

The model parameters are likely to vary from cell to cell. This will be the case even for a particular cell type and when cell size is taken into account. To determine whether this affects our results, we used a GPU to run our simulations for a range of parameter sets [39]. This allowed us to efficiently check hundreds of thousands of different parameter choices. In particular, we chose to vary the four single channel conductances (*g*_1,Ca_, *g*_1,K_, *g*_1,SK_, *g*_1,BK_), the leak conductance and reversal potential (*g*_*l*_, *V*_*l*_), and the calcium extrusion rate *k*_*c*_. For each simulation, we picked each parameter randomly from a uniform distribution with a range ±50% of the parameter’s default value.

Each parameter set can then be classified into one of four basic types—depolarised, hyperpolarised, noisy steady state or event-containing—based on the behaviour of the membrane potential *V* (once the initial transient period has passed). The depolarised and hyperpolarised cases are defined by the size of variations in *V* being less than 10 mV, with depolarised (hyperpolarised) corresponding to whether the mid-range of *V* is above (below) −50 mV. Noisy steady states encompass a number of different behaviours: (i) cases with no events, (ii) cases where the range of *V* lies between 10 mV and 35 mV, and (iii) cases where the average event duration is much longer than the average inter-event time. All other cases are labelled as event-containing. We further classify the event-containing cases as either pure spiking (BF = 0), almost pure spiking (0<BF<0.05), pure bursting (BF = 1), almost pure bursting (0.95<BF<1) or mixed (0.05≤BF≤0.95). See S1 Text for full details.

Based on 700,000 parameter sets, we find that 92.4% contain events, with only 0.1% depolarised, 3.0% hyperpolarised and 4.5% noisy steady states. Of those that contain events, 3.9% correspond to pure spiking, 5.8% to almost pure spiking, 6.0% to pure bursting, 6.8% to almost pure bursting, and 77.5% to mixed cases.

We now examine the sigmoidal behaviour of BF against *N*_BK_. Above, we found that BF increases with *N*_BK_ (with *g*_1,BK_ fixed) but typically saturates before reaching pure bursting (Fig 2). We can now test whether this is also the case for other parameter choices. For each randomly-chosen parameter set, we consider a range of values for *N*_BK_ (with a corresponding change in *g*_BK_). We only study cases where all choices of *N*_BK_ are event-containing and where there is a large enough BF range to make comparisons meaningful. For example, cases where nearly all *N*_BK_ values corresponded to pure spiking were not included. Of the selected parameter sets, over 98% (141,449 of 143,870) exhibit the sigmoidal behaviour shown in Fig 2. Note that we allow small decreases in BF for large *N*_BK_ as happens in Fig 2. Further, we find that the large *N*_BK_ limit of BF is usually less than unity, with an average of 0.80 and a standard deviation of 0.12. Thus, for most parameter choices, even very large numbers of BK channels do not lead to pure bursting.

The few cases where BF does not increase with *N*_BK_ are interesting to study, and there are even cases where BF decreases with increasing *N*_BK_. For example, this occurs if the maximal conductance for Ca channels is increased to *g*_Ca_ = 3.5 nS and that for K channels decreased to *g*_K_ = 1.8 nS. In the deterministic model, this parameter choice produces a depolarised steady state for *N*_BK_ < 18 and pure bursting for larger *N*_BK_. However, the inclusion of channel noise causes small *N*_BK_ to now correspond to pure bursting, with BF gradually dropping to around 0.4 for large *N*_BK_.

Finally, we examine our result that larger cells exhibit more bursting. Again we choose random parameter sets, but now compare three cell sizes (λ = 0.5, 1.0 and 1.5) at two different channel densities (corresponding to *N*_BK_ = 4 and *N*_BK_ = 20 when λ = 1.0). As before, we neglect cases where not all simulations contain events. Of the remaining cases, 89.8% (144,670 of 161,116 parameter sets) for the first channel density and 99.7% (153,781 of 154,272) for the second show BF increasing with cell size.

## Discussion

Here, by explicitly modelling the stochastic opening and closing of ion channels, we have been able, for the the first time, to study the effect of realistic channel noise in a model of the electrical activity of endocrine cells in the anterior pituitary. We have shown that noise plays an important role in spiking and bursting activity, not least by allowing individual cells to display both spiking and bursting behaviour. In particular, we were able to determine how channel noise determines the ratio of bursting to spiking activity, which can be quantified by the bursting fraction (BF).

We found that different numbers of BK channels lead to different bursting fractions, with more channels typically causing more bursting. This is directly opposite to the result for K and SK channels, where increasing the number of channels reduces BF. We also found that, even for large numbers of BK channels (and so large total BK conductances), pure bursting is rarely obtained. Instead cells always exhibit some spiking. This is in stark contrast to a previous model (which used a normally distributed random noise current) [16] and shows that including realistic channel noise is essential to fully understand this system.

Our results complement previous work on how ion channel stochasticity affects electrical activity in neurons and pancreatic *β*-cells [22–25]. For example, channel noise affects the reliability of spike timing in response to stimuli, and smoothens the spiking frequency response to various levels of input currents. In an otherwise silent model, channel fluctuations can even provoke action potentials if channel density is low enough [6, 20]. While most studies have focused on the statistics of interspike intervals, some work have shown that, in neurons, channel flicker is essential for subthreshold oscillations [40] and can blur the transition between spiking and bursting [21].

A particular advantage of our model of endocrine pituitary cells is that it allows us to determine the relative effect of noise in different channel types. This reveals that, because of their relatively small number and large single-channel conductance, it is the stochastic opening and closing of BK channels that accounts for almost all the BF-versus-*N*_BK_ behaviour (Fig 4C). Whereas noise in other channels is the source of most of the small fluctuations in the membrane potential, the difference in *V*_max_ between spikes and bursts is largely due to noisy BK channels.

In addition, by perturbing individual channels, we were able to study how noise can cause a would-be spike to become a burst and vice versa. This can occur with the opening or closing of only a single BK or K channel, but needs more than 40 Ca or SK channels to be simultaneously opened or closed (a much less common scenario). Further, fluctuations in K or Ca channels only convert between spikes and bursts when the fluctuation occurs after *V*_max_. This is not the case for BK channels, where fluctuations at any point can have a significant effect.

Perhaps the most important application of our model is to the effect of cell size on electrical activity. Even in the deterministic model, this leads to interesting, nontrivial predictions, such as spiking/bursting being associated with small/large cells. This can be understood, at least within the model, as due to effectively slower calcium dynamics in larger cells, which results in longer events. Moreover, the full stochastic model shows that, not only do larger cells burst more, but that bursting starts at lower *g*_BK_ densities in larger cells. In addition, our model predicts that the smallest cells never exhibit much bursting: around half of events remain spikes even for very large numbers of BK channels.

That slower calcium kinetics tend to promote bursting has been demonstrated experimentally using intracellular calcium buffering in cerebellar granule cells [41]. A relationship between cell size and tendency to burst has also been shown previously in a model of dopamine neurons, where dendrites and soma were increased by different factors to match the ratio of sizes between human and rat neurons [42]. Perhaps not surprisingly, this means that larger cells, with longer event durations, typically release more hormone than smaller cells [43].

Understanding the stochastic nature of this system allows us to start to appreciate how cells, by controlling spiking and bursting behaviour, could fine-tune levels of hormone release. In particular, it is conceivable that the number and/or distribution of BK channels could be adjusted during the cell lifetime in order to modulate the bursting fraction. Perhaps even a population of different cell sizes and parameters could allow a graded response to the required hormone levels. For example, certain parameter choices lead to a situation where there is a minimum level of bursting for all cell sizes: every cell, whatever the size, is guaranteed to have at least this minimum fraction of bursts (Fig 6B). Similarly, it is possible to ensure a maximum level of bursting (for any number of BK channels) simply by choosing either a small cell size (Fig 6C) or a large BK time constant (Fig 3C). Of course, it is important to remember that the bursting fraction is only one aspect of this system: endocrine cells are likely to have evolved to address a range of other factors, such as the speed of response to extracellular signals and robustness to external perturbations.

It is important to point out the assumptions and potential limitations of our model. First, although we have included stochastic potassium and calcium channels, we have not considered noise in the leak current or the intracellular calcium concentration. Second, we have used a two-state channel model (each channel can only be either completely open or completely closed). Although more detailed models that include the whole sequence of states visited by ion channels could be implemented [44], this will not change the importance of BK channels, which stems from their scarcity and relatively large single channel conductance. Third, we have assumed that channels open and close independently of both their environment (*e.g*. other nearby channels) and their own history (*e.g*. the time since they last switched state). This assumption is likely to be only approximately true. Fourth, we have modelled BK channels as gated only by voltage, ignoring any influence by nearby Ca^2+^ channels. Recent work has started to address this issue, which could be incorporated into future versions of our model [45, 46].

We have also assumed that the number of channels per cell is proportional to the cell membrane area. Although this is a sensible first approximation, the exact relationship may not be so straightforward. For example, it is possible that only a certain fixed number of channels are synthesised per cell, independent of the cell size. Conversely, a larger cell may need to increase channel density (and thus gene expression) in order to maintain an appropriate intracellular calcium concentration [47].

It may at first appear surprising that the number of BK channels in pituitary cells is so small, especially since Van Goor et al. have reported BK currents in somatotrophs of 4 nA at *V* = + 90 mV [9], which (assuming a potassium reversal potential of −75 mV) corresponds to a conductance of just over 24 nS, *i.e*. to about 240 BK channels. However, these measurements were made using long pre-pulses to maximise the intracellular Ca^2+^ concentration. At normal Ca^2+^ levels, during an action potential, only the BK channels closely associated to Ca^2+^ channels will sense enough Ca^2+^ to be activated [28]. The fast BK current resulting from a depolarising step from −40 mV to + 40 mV varies between 40 and 120 pA [16], which corresponds to activation of only between 5 and 15 BK channels.

There are a number of ways that our work here could be extended in future, particularly with regard to classification of events. We have identified all events as either spikes (events with short duration and no oscillations in the depolarised state) or bursts (events with long duration and/or oscillations). A future approach could consider more fine-grained categories with, for example, bursts split into short bursts (short duration events with oscillations) and long bursts (long duration events). Long bursts could even be split into two classes depending on whether oscillations are present. Such an approach would lead to a number of different bursting fractions (BFs) corresponding to the different types of bursts. Yet another approach could dispense with the bursting fraction altogether and focus instead on the average event duration. Finally, rather than considering single cells with fixed parameters, it may be fruitful to model a whole population of cells with a range of sizes and channel numbers. This would avoid the channel rounding problem where a single cell cannot represent a non-integer number of channels.

Our modelling here has generated a number of unexpected predictions that could readily be tested experimentally by pharmacologically blocking channels and adding artificial currents using dynamic clamp [48]. For example, by blocking and re-introducing BK currents, it would be possible to check both whether very large BK conductances rarely lead to pure bursting (Fig 2) and whether (for some fixed values of *g*_BK_) the greatest proportion of spikes corresponds to an intermediate number of BK channels (Fig 3A). It should also be possible to check the effect of channel perturbations by injecting short current bursts at particular time points during events (Fig 7). In addition, the role of cell size could be examined by selecting cells of various sizes. By this means it would be possible to check our predictions that (i) larger cells tend to burst more, (ii) bursting first appears at smaller *g*_BK_ densities in larger cells, (iii) small cells rarely produce a large fraction of bursts even for very large BK conductances, and (iv) the lowest bursting fractions occur in small (but not the smallest) cells.

It is no longer possible to sustain the old-fashioned view that noise is an unfortunate and undesirable property of real-world systems that is best neglected. Nowhere is this more apparent than in cell biology, where noise is an integral, and often useful, component. As we have shown here, the stochastic opening and closing of ion channels plays a fundamental role in the behaviour of endocrine cells in the anterior pituitary, leading to surprising results concerning the types of channels involved and the effect of cell size. We expect future work to continue to discover novel aspects of noise in membrane electrical activity, not just in the pituitary gland, but in other related excitable cellular systems.

## Methods

### Parameter values

Unless otherwise stated, we use the following parameter values [16]. *C* = 10 pF, *g*_Ca_ = 2 nS, *g*_K_ = 3.2 nS, *g*_SK_ = 2 nS, *g*_BK_ = 0.5 nS, *g*_*l*_ = 0.2 nS, *V*_Ca_ = 60 mV, *V*_K_ = −75 mV, *V*_*l*_ = −50 mV, *τ*_*m*_ = 0.1 ms, *τ*_*n*_ = 30 ms, *τ*_*s*_ = 0.1 ms, *τ*_BK_ = 5 ms, *v*_*m*_ = −20 mV, *s*_*m*_ = 12 mV, *v*_*n*_ = −5 mV, *s*_*n*_ = 10 mV, *v*_*f*_ = −20 mV, *s*_*f*_ = 2 mV, *k*_*s*_ = 0.4 *μ*M, *f*_*c*_ = 0.01, *α* = 0.0015 *μ*M/fC, *k*_*c*_ = 0.12 ms^−1^.

### Estimation of channel numbers

The number of channels is likely to depend on the cell size. For simplicity we assume that, for a given channel type, there is a constant number of channels per unit membrane area. In particular, for concreteness, we take a typical cell to have diameter 10 *μ*m and contain 200 Ca channels, 640 K channels, 200 SK channels and 5 BK channels. These are based on literature values for the single channel conductances of *g*_1,Ca_ = 10 pS, *g*_1,K_ = 5 pS, *g*_1,SK_ = 10 pS and *g*_1,BK_ = 100 pS [49, 50]. We have checked that our conclusions are unchanged if these values are decreased by up to 50% or increased by up to 100% (see S1 Text).

### Numerical simulations

The system was solved numerically using the Euler method with step size Δ*t* = 0.01 ms. Each time step involved (i) calculating the currents (Eq (2)), (ii) applying any perturbation, (iii) updating *V* and [Ca] (Eqs (1) and (5)), (iv) calculating the steady-state activation functions (Eq (4)), and (v) stochastically determining how many channels open and close during the time step. We checked (for a range of parameter values) that smaller values of Δ*t*, down to Δ*t* = 1 × 10^−4^ ms, do not lead to noticeably different results. For full details, see S1 Text.

## Supporting information

S1 Text Further details of the mathematical model, the numerical simulations and the GPU simulations. (PDF)
