# Supplementary material for: Ion channel noise shapes the electrical activity of endocrine cells
Source: PLoS Comput Biol. 2020 Apr 6;16(4):e1007769. doi: 10.1371/journal.pcbi.1007769 (PMC7162531; doi:10.1371/journal.pcbi.1007769)
Supplement: S1 Text — (PDF) [file pcbi.1007769.s001.pdf]

Supporting Text for *Ion channel noise shapes the electrical activity of endocrine cells*

David M. Richards[1,*], Jamie J. Walker[2], Joel Tabak[3]

[1] Living Systems Institute, University of Exeter, Exeter, EX4 4QD, UK
[2] College of Engineering, Mathematics and Physical Sciences, University of Exeter, Exeter, EX4 4QF, UK
[3] University of Exeter Medical School, University of Exeter, EX1 2LU, Exeter, UK

* E-mail: david.richards@exeter.ac.uk

Contents

1 Further details of the mathematical model **2**
- 1.1 The deterministic model 2
- 1.2 The stochastic model 2
- 1.3 The average and standard deviation of the number of open channels 3

2 The number of channels **4**

3 Numerical simulations **4**
- 3.1 Details of the numerics 4
- 3.2 Event identification 5

4 Details of cell size dependence **6**
- 4.1 Parameter scalings 6
- 4.2 Equivalent scalings 8
- 4.3 Scaling of the calcium dynamics 8

5 Further details of perturbations **10**

6 Details of GPU simulations **10**

7 Phase plots **12**
- 7.1 Changing the cell size 12
- 7.2 The effect of perturbations 12

1 Further details of the mathematical model

1.1 The deterministic model

Our model is based on the model by Tabak et al. [1], with two new variables that describe the gating variables for the Ca and SK channels. This results in a six variable model—the membrane potential $V(t)$, the Ca, K, SK and BK gating variables $m(t)$, $n(t)$, $s(t)$, $f(t)$, and the intracellular calcium ion concentration [Ca](t)—described by Eqs. (1-5) in the main text.

Initial conditions are taken as $V(0) = -60\,\mathrm{mV}$, $m(0) = m_\infty(V(0))$, $n(0) = 0.1$, $s(0) = s_\infty([\mathrm{Ca}](0))$, $f(0) = 0.01$ and $[\mathrm{Ca}](0) = 0.27\,\mu\mathrm{M}$. Simulations are run for long enough that the initial transient period has little effect on our results.

It is worth noting that it is sometimes more convenient to write the currents (Eq. (2) in the main text) in terms of the single channel conductances ($g_{1,\mathrm{X}}$) and the number of open channels ($N_{\mathrm{op},\mathrm{X}}$):

$$\begin{aligned} I_{\mathrm{Ca}} &= g_{1,\mathrm{Ca}} N_{\mathrm{op,Ca}}(V - V_{\mathrm{Ca}}), \quad I_{\mathrm{K}} = g_{1,\mathrm{K}} N_{\mathrm{op,K}}(V - V_{\mathrm{K}}), \\ I_{\mathrm{SK}} &= g_{1,\mathrm{SK}} N_{\mathrm{op,SK}}(V - V_{\mathrm{K}}), \quad I_{\mathrm{BK}} = g_{1,\mathrm{BK}} N_{\mathrm{op,BK}}(V - V_{\mathrm{K}}). \end{aligned} \tag{S1}$$

1.2 The stochastic model

Consider channel X (which can be either Ca, K, SK or BK), with gating variable x. The dynamics of x are given by (cf. Eq. (3) in the main text)

$$\tau_x \frac{dx}{dt} = x_\infty - x, \tag{S2}$$

where x_∞ depends on either V or [Ca], τ_x is the time constant, and $0 \leq x \leq 1$. This can be rewritten as

$$\frac{dx}{dt} = \beta(1 - x) - \gamma x, \tag{S3}$$

where $\beta = x_\infty/\tau_x$ and $\gamma = (1 - x_\infty)/\tau_x$. Both β and γ satisfy $0 \leq \beta, \gamma \leq \frac{1}{\tau_x}$.

We can now reinterpret this equation as the master equation for the probability $P_{\mathrm{op}} = x$ that a single channel is open. Clearly $(1 - x)$ is then the probability P_{cl} that the same channel is closed so that, with more standard notation, our master equation is

$$\frac{dP_{\mathrm{op}}}{dt} = \beta P_{\mathrm{cl}} - \gamma P_{\mathrm{op}}. \tag{S4}$$

This can now be seen as arising from a two-state Markov process with non-constant transition rates (since β and γ are functions of either V or [Ca]).

Rather than a single channel, our system is such that we have a (finite) total of N_{X} channels of type X. Of these, at any given time, N_{op} are open and $N_{\mathrm{cl}} = N_{\mathrm{X}} - N_{\mathrm{op}}$ are closed. Then the gating variable x describes the

proportion of open channels, $x = N_{\rm op}/N_{\rm X}$. Note that the value of x can now only take a finite number of discrete values: 0, $1/N_{\rm X}$, $2/N_{\rm X}$, $3/N_{\rm X}$, ..., 1. Instead of $N_{\rm X}$ separate Markov processes, we can consider a single Markov process for the number of open and closed channels:

$$N_{\rm cl} \underset{\gamma}{\overset{\beta}{\rightleftarrows}} N_{\rm op}. \tag{S5}$$

There are various ways in which such a Markov process could be simulated. The one employed here is to use a sufficiently small time step Δt such that $\beta\Delta t$ and $\gamma\Delta t$ can be considered the probabilities that a given closed/open channel opens/closes in one time step. This can only make sense if both probabilities are sufficiently small ($\beta\Delta t \ll 1$ and $\gamma\Delta t \ll 1$). This ensures that (on average) only a few of the $N_{\rm X}$ channels open or close each time step. If this is not the case, then the probability that a given (say) closed channel opens and then recloses in a single time step cannot be ignored. Similarly, in such a case it would also be necessary to take into account more complex possibilities such as channels that open, close and reopen all within one time step. Accurate simulations that consider such possibilities and allow multiple channel state switching within a given fixed time step (such as τ-leaping), or which avoid the issue by using continuous time (as with the Gillespie stochastic simulation algorithm (SSA)), are possible but are not necessary here [2].

Since $0 \leq x_\infty \leq 1$, the requirement of small $\beta\Delta t$ and $\gamma\Delta t$ implies that the time step must be chosen sufficiently small. In particular, we need $\Delta t \ll \tau_x$ for all channel types. Assuming short enough Δt, any single open channel, after one time step, closes with probability (approximately) $\gamma\Delta t$ and remains open with probability $1 - \gamma\Delta t$. For $N_{\rm op}$ open channels (assuming that each channel opens and closes independently of the others), this means that the number of open channels that close in one time step follows the binomial distribution $B(N_{\rm op}, \gamma\Delta t)$. The situation is similar for the closed channels, with the number opening per time step described by the binomial distribution $B(N_{\rm cl}, \beta\Delta t)$. Again, it is important to emphasise that simulating by sampling from a binomial distribution is only accurate if the probability of a given channel opening or closing each time step is sufficiently small.

1.3 The average and standard deviation of the number of open channels

For x_∞ and τ_x fixed (corresponding to constant V and [Ca] without any time dependence), the number of open channels follows a binomial distribution [3] with average

$$\langle N_{\rm op}\rangle = x_\infty N_{\rm X} \tag{S6}$$

and standard deviation

$$\delta N_{\rm op} = \sqrt{x_\infty(1-x_\infty)N_{\rm X}}. \tag{S7}$$

The coefficient of variation (*i.e.* the relative standard deviation), which is often a more useful measure of spread, is given by

$$\frac{\delta N_{\rm op}}{\langle N_{\rm op}\rangle} = \sqrt{\frac{1/x_\infty - 1}{N_{\rm X}}}. \tag{S8}$$

The number of closed channels also follows a binomial distribution with $\langle N_{\rm cl}\rangle = (1-x_\infty)N_{\rm X}$ and $\delta N_{\rm cl} = \delta N_{\rm op}$. It is worth nothing that, although τ_x appears in the state switching probabilities $\beta\Delta t$ and $\gamma\Delta t$, it does not appear in these steady-state quantities (which only depend on the ratio of β to γ); τ_x only affects the speed at which $N_{\rm op}$ and $N_{\rm cl}$ move towards their average values.

Finally, the number of open (and closed) channels in the full model will typically only be approximately binomially distributed. This is because (unless we are in a steady state) V and [Ca] (and so x_∞) are continually changing. This means that β and γ are not fixed and so the steady-state distributions of $N_{\rm op}$ and $N_{\rm cl}$ are never reached.

2 The number of channels

Our estimates for the number of channels are based on measured single channel conductances and total conductances. We assume that the measured total conductances apply to a "typical" cell of diameter $10\,\mu$m. Then from $g_{1,\rm Ca} = 10\,$pS, $g_{1,\rm K} = 5\,$pS, $g_{1,\rm SK} = 10\,$pS, $g_{1,\rm BK} = 100\,$pS and $g_{\rm Ca} = 2\,$nS, $g_{\rm K} = 3.2\,$nS, $g_{\rm SK} = 2\,$nS, $g_{\rm BK} = 0.5\,$nS [1, 4, 5], we estimate that $N_{\rm Ca} = 200$, $N_{\rm K} = 640$, $N_{\rm SK} = 200$ and $N_{\rm BK} = 5$.

However, even if these values are not accurate, we have checked that our results are unchanged. In particular, we checked the consequences of our estimated values of $N_{\rm X}$ being incorrect by up to a factor of two (*i.e.* we considered the effect of setting $N_{\rm Ca} = 100$, $N_{\rm Ca} = 400$, $N_{\rm K} = 320$, $N_{\rm K} = 1280$, $N_{\rm SK} = 100$ or $N_{\rm SK} = 400$). In each case we checked that (i) BK still increases with $N_{\rm BK}$ (for fixed $g_{1,\rm BK}$) and (ii) BF still increases with cell size. In fact, we expect that our conclusions are still likely to hold even for larger changes in channel number.

3 Numerical simulations

3.1 Details of the numerics

The deterministic part of our model was solved numerically using the forward Euler method and a step size of $\Delta t = 0.01\,$ms. This involved calculating the currents (Eq. (2) in the main text) and then updating V and [Ca]

based on finite difference versions of Eqs. (1) and (5). Most simulations where run for a simulation time of $t_{\max} = 2{,}000\,\mathrm{s}$.

The stochastic opening and closing of channels was implemented as follows. For each channel type, at each time step, n_1 of the N_{op} open channels were closed, where n_1 was chosen from a binomial distribution $B(n, p)$ with $n = N_{\mathrm{op}}$ and $p = \gamma\Delta t$. Similarly n_2 of the N_{cl} closed channels were opened with n_2 drawn from a binomial distribution with $n = N_{\mathrm{cl}}$ and $p = \beta\Delta t$. Thus, at each time step,

$$N_{\mathrm{op}} \to N_{\mathrm{op}} - n_1 + n_2, \tag{S9}$$

where $n_1 \sim B(N_{\mathrm{op}}, \gamma\Delta t)$ and $n_2 \sim B(N_{\mathrm{cl}}, \beta\Delta t)$. Of course, N_{cl} is not independent, but always given by $N_{\mathrm{X}} - N_{\mathrm{op}}$. Finally, the relevant gating variable was calculated as $N_{\mathrm{op}}/N_{\mathrm{X}}$.

As explained above, this equation for the change in N_{op} is only accurate if both $\beta\Delta t$ and $\gamma\Delta t$ (and hence the averages of n_1 and n_2) are small. This ensures that a given closed (open) channel is highly unlikely to open (close) and then reclose (reopen) all within one time step. This in turn requires that the time step Δt is sufficiently small (compared to the relevant time constant τ_x). For our time step of $\Delta t = 0.01\,\mathrm{ms}$, we explicitly checked that only a few channels opened/closed each time step for the range of parameter values considered in this work. Further, we also checked that reducing Δt to $0.001\,\mathrm{ms}$ or $0.0001\,\mathrm{ms}$ led to no noticeable differences in any of our output variables (particularly BF).

3.2 Event identification

Events are defined as periods when the membrane potential is sufficiently depolarised, in particular periods when V lies above some fixed threshold V_{event}. An event starts when V rises above V_{event} and ends when V drops back below V_{event}. Various other definitions are possible, such as allowing the threshold to depend on the model parameters or using separate thresholds for the start and end of events. Although the definition of events will always have a subjective quality, we believe that all sensible definitions are likely to lead to similar conclusions.

To avoid certain pathological cases, we introduce a minimum time gap t_{gap} between the starts and ends of events. The case when V rises above V_{event} and then quickly drops below again (before t_{gap} has elapsed) is not classified as an event. Similarly, the case where, during an event, V momentarily drops below V_{event} for less than t_{gap} is counted as only one event rather than two. Thus, the algorithm to identify events must deal with the following four situations:

Threshold direction	Time since last event start/end	Description	Action	Sketch
V crosses V_{event} from below	Time since end of last event $\geq t_{\text{gap}}$	Normal start of event	Start new event	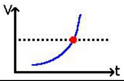
	Time since end of last event $< t_{\text{gap}}$	Previous event just finished	Continue previous event	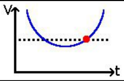
V crosses V_{event} from above*	Time since start of event $\geq t_{\text{gap}}$	Normal end of event	End event	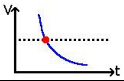
	Time since start of event $< t_{\text{gap}}$	Event has just started	Cancel event	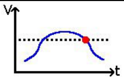

Some events contain oscillations of V within the depolarised state. To quantify this, we count the number of peaks in V during each event. This involves introducing a new parameter, V_{min}, which specifies the minimum prominence for a maximum to be counted as a peak.

Each event is classified into one of two subtypes—spikes or bursts—with the difference based on event duration and number of peaks. The role of event duration is controlled by t_{spike}. Spikes are defined as events with duration less than t_{spike} and only one peak (*i.e.* without oscillations). All other events are classified as bursts, which therefore include both events lasting longer than t_{spike} and shorter events that contain at least one oscillation.

Although we here only consider spikes and bursts, other categories of events could also be defined. For example, it may be useful to distinguish short bursts (events with duration less than t_{spike} and more than one peak) from long bursts (events with duration longer than t_{spike}). Long bursts could further be split into those with and without oscillations.

Throughout this paper, we use the following values for the event identification parameters: $V_{\text{event}} = -45\,\text{mV}$, $t_{\text{gap}} = 10\,\text{ms}$, $V_{\text{min}} = 2\,\text{mV}$, $t_{\text{spike}} = 100\,\text{ms}$.

4 Details of cell size dependence

4.1 Parameter scalings

We take a cell with diameter $2R = 10\,\mu\text{m}$ as the "standard" cell and consider scaling the radius R by a factor λ. Then the surface area and volume scale with λ^2 and λ^3 respectively. It is likely that many parameters will be unchanged by this scaling. This applies to the reversal potentials (V_{Ca}, V_{K}, V_l), time constants (τ_m, τ_n, τ_s, τ_{BK}), voltage midpoints (v_m, v_n, v_f),

*The transition from above to below V_{event} can also occur if the initial value of V lies above the threshold, *i.e.* $V(0) > V_{\text{event}}$. This is not related to any event and is ignored until V crosses back over the threshold, triggering the start of the first event.

calcium midpoint (k_s), slope parameters (s_m, s_n, s_f), and free-to-bound calcium fraction (f_c).

Other parameters are expected to depend on cell size. First, consider the channel numbers (N_{X}). For each channel type, we assume there is some approximately constant area density of channels within the membrane so that the channel numbers scale with membrane area. It is important to remember that this is only an assumption and warrants further verification. For example, it is possible that, at least for some channel types, only a fixed number of channels are synthesised per cell, independent of cell size. Equally, it is also possible that, since channels are produced within the cytoplasm, their number scales more like the cell volume.

Second, the membrane capacitance (C) and maximum conductances (g_{Ca}, g_{K}, g_{SK}, g_{BK}, g_l) are expected to scale with membrane area, *i.e.* as λ^2. This is because the capacitance is a measure of how much charge the membrane can hold per unit voltage and the conductances are assumed to be proportional to the number of channels. As a consequence of this, for fixed V and gating variables, the currents all scale as $I_{\mathrm{X}} \to \lambda^2 I_{\mathrm{X}}$.

Finally, we consider the α and k_c parameters. Since [Ca] is a molar concentration, α (the conversion factor from charge to concentration) scales like the inverse of the volume ($1/\lambda^3$). Calcium extrusion is normally via the cell membrane (through dedicated exchangers such as NCX) and yet affects the concentration throughout the whole cytoplasm. This suggests that the extrusion rate k_c should scale like area over volume, *i.e.* like $1/\lambda$. This assumes that calcium "extrusion" is purely a cell membrane effect. If, for example, calcium was also/instead removed via sequestration by some cytoplasmic complex, then this scaling behaviour would need modifying. Similarly, if uptake of calcium into the ER and mitochondria is considered, this could further complicate the situation. For example, the number of mitochondria (and hence their total surface area) might be expected to scale with cell volume rather than with the cell membrane area. The ER may have even more complicated scaling behaviour due to its structure as a network of cisternae.

Thus, in summary, a scaling of the cell radius by $R \to \lambda R$ requires the following parameter changes.

Parameter	Scaling
Membrane capacitance C	$C \to \lambda^2 C$
Conductances g_{X}	$g_{\mathrm{X}} \to \lambda^2 g_{\mathrm{X}}$
Leak conductance g_l	$g_l \to \lambda^2 g_l$
Charge to molar conversion, α	$\alpha \to \alpha/\lambda^3$
Ca^{2+} extrusion rate, k_c	$k_c \to k_c/\lambda$
Number of channels, N_{X}	$N_{\mathrm{X}} \to \lambda^2 N_{\mathrm{X}}$

Since the number of channels must always be an integer, it is necessary

to either (i) only choose values of λ for which all $N_{\rm X}$ are integers, or (ii) round $N_{\rm X}$ after scaling. Further, we need to ensure that $N_{\rm X} \geq 1$ for all channel types. This places a lower limit on λ since cells that are too small will not contain, for example, even a single BK channel.

The second option, where the $N_{\rm X}$ are rounded after scaling, can often introduce "unevenness" in the channel number. For example, with $\lambda = 0.5$ and the number of channels as above, $N_{\rm BK}$ needs to be rounded (from 1.25 down to 1) whereas the other $N_{\rm X}$ are already integers. Of course, this unevenness is not necessarily unrealistic since real cells may well face the same problem. One possible solution could be, for each λ, to consider a population of cells with a range of different $N_{\rm X}$. For example, for $\lambda = 0.5$, the population could involve three cells with $\{N_{\rm Ca}, N_{\rm K}, N_{\rm SK}, N_{\rm BK}\} = \{50, 160, 50, 1\}$ and one cell with $\{N_{\rm Ca}, N_{\rm K}, N_{\rm SK}, N_{\rm BK}\} = \{50, 160, 50, 2\}$.

4.2 Equivalent scalings

Since the time constants and the steady-state activation function parameters are unchanged by scaling the radius, the equations for the four gating variables (Eq. (3) in the main text) are also unchanged. This is also the case for V (Eq. (1) in the main text) where the cell size dependence drops out (both the capacitance C and currents $I_{\rm X}$ scale like λ^2). However, the [Ca] equation (Eq. (5) in the main text) is scaled by $1/\lambda$ on the right-hand side. This means that the overall effect of increasing/decreasing the cell radius is effectively to slow down/speed up the calcium dynamics whilst leaving the other dynamics unchanged.

Because of this, within the model, the scaling $R \to \lambda R$ is equivalent to simply scaling $f_c \to f_c/\lambda$ and $N_{\rm X} \to \lambda^2 N_{\rm X}$. This does not mean that f_c (the fraction of calcium ions that are unbound) actually changes with cell size, simply that this scaling gives the same dynamics for the six model variables (V, m, n, s, f, [Ca]). In particular, under this equivalent scaling, the currents do not scale correctly and no longer correspond to the real physical currents. Similarly, the maximum conductances scale incorrectly, with the consequence that the single channel conductances appear to scale as $g_{1,{\rm X}} \to g_{1,{\rm X}}/\lambda^2$. Again, this is not a real physical effect: the behaviour of individual channels does not depend on cell size. Because of these subtleties, and to avoid possibly confusion, we do not use this equivalent scaling in our simulations.

4.3 Scaling of the calcium dynamics

So far, we have assumed a particular scaling of the calcium dynamics (described by Eq. (5) in the main text) where $\alpha \to \alpha/\lambda^3$ and $k_c \to k_c/\lambda$. This is based on the idea that the dynamics of [Ca] occur throughout the whole cell volume. As a consequence, the right-hand side of Eq. (5) (and so also $\frac{d[{\rm Ca}]}{dt}$)

scales with $1/\lambda$, meaning that larger cells have effectively slower calcium dynamics.

However, this is an assumption that is likely to be only approximately correct. It is instead possible that most of the changes in [Ca] take place only in a thin shell adjacent to the cell membrane (close to where the calcium channels are). Assuming this shell has constant thickness, then the shell volume (*i.e.* the volume where the calcium dynamics takes place) would scale with the cell surface area (*i.e.* with λ^2) rather than with cell volume.

This would change the scaling of the calcium parameters to $\alpha \to \alpha/\lambda^2$ and $k_c \to k_c$, so that Eq. (5) would no longer have any dependence on λ. Although in the deterministic case this would mean that our model (and so the dynamics of V and [Ca]) would not depend on cell size, this would not be the case in the full stochastic model, where the number of channels would still scale as $N_{\mathrm{X}} \to \lambda^2 N_{\mathrm{X}}$. Thus, even if the calcium dynamics are confined to a thin submembrane shell, cell size would still play an important role and affect BF. In particular, larger cells would have more channels and so would be closer to the deterministic model. We have already examined this scenario in the "The number of channels and the BK time constant" section with the results summarised in Fig. 3A. Based on this, the model would then predict that whether large cells burst more or less than smaller cells would depend crucially on the exact parameter choices. This in turn would suggest that there may be two distinct populations of endocrine cells: one where bursting becomes more common with increasing cell size and one where instead spiking is more dominant in larger cells.

To understand this issue properly, and so determine whether the volume where the calcium dynamics takes place scales with λ^2 or λ^3, requires a full spatio-temporal model of the calcium dynamics that includes the intracellular spatial distribution of Ca^{2+}. Preliminary work in this direction (for endocrine cells) has been initiated by Tagliavini et al. [6]. This shows that (at least in endocrine cells) the free calcium concentration varies considerably even at substantial distances from the cell membrane (well over $1.5\,\mu$m), which suggests that the λ^3 scaling may be more appropriate for endocrine cells. Interestingly, this may not be the case for other cell types. For example, the calcium concentration in neurons, which are typically much larger (especially when dendrites, axons and axon terminals are taken into account), may vary much less in the cell interior so that λ^2 scaling may be the more accurate description.

The question of exactly how the relevant volume for calcium dynamics scales with cell size is complex. The actual scaling is likely to lie somewhere between λ^2 and λ^3 and can probably not be represented simply as a power of λ. This situation is further complicated by uptake of calcium into mitochondria and the ER as discussed above. The current work is likely to be only a first step in determining the role of cell size in the electrical activity of endocrine cells. A future, more detailed diffusion model that includes the

spatial distribution of calcium will be needed to shed further light on this topic.

5 Further details of perturbations

To study the effect of the stochastic opening and closing of channels, we sometimes manually perturb the system at particular points during events. This is implemented in one of two ways, either by manually opening/closing particular channels or by injecting a current for a short period of time. The perturbation is triggered by a membrane potential threshold V^* with, for each event, the perturbation imposed either the first time V rises above V^* or the first time V drops below V^*. Although V can cross V^* multiple times during each event (*e.g.* during bursts or due to noise), we only apply the perturbation the first time this occurs, ensuring at most one perturbation per event.

For the stochastic model, we choose to perturb by manually opening or closing a given number of channels. This allows us to determine how fluctuations in individual channels can convert spikes into bursts and vice versa. This involves artificially increasing or decreasing N_{op} for a given channel type. In each case we ensure that, after the perturbation, $0 \leq N_{\text{op}} \leq N_{\text{X}}$ (*i.e.* that $0 \leq x \leq 1$).

In the deterministic model it is not possible to implement perturbations in the same way. This is because, at least for channels with small time constants (Ca and SK), any perturbation quickly disappears within only a few time steps, resulting in practically no difference to the unperturbed case. One solution is to lock the gating variables for some fixed time (*e.g.* 5 ms) after the perturbation. For example, if a perturbation increases x then, for the next 5 ms, x would be prevented from decreasing (although increase is still possible). Although we initially tried this method, we later switched to using an injected current as the perturbation. For a perturbation in channel X, this involved adding a current $\pm g_{\text{X}} x(V - V_{\text{X}})$ for 5 ms with $x = q/N_{\text{X}}$ and q representing the number of channels opened or closed. Note, this is not a constant current since V typically changes at each time step.

6 Details of GPU simulations

To check how sensitive our results are to the model parameters, we ran our simulations for a range of randomly chosen parameter sets. In particular, we varied seven parameters: the four single channel conductances ($g_{1,\text{Ca}}$, $g_{1,\text{K}}$, $g_{1,\text{SK}}$, $g_{1,\text{BK}}$), the two leak current parameters (g_l, V_l), and the calcium extrusion rate (k_c). Before each simulation, we randomly chose values for these seven parameters and used these values throughout the entire simulation. Each parameter was chosen from a uniform distribution with range

±50% of the usual value. For example, the single channel BK conductance was chosen so that $50\,\mathrm{pS} \leq g_{1,\mathrm{BK}} \leq 150\,\mathrm{pS}$.

To speed up testing many parameter sets, we converted our code (using the CUDA platform for C) to run on a GPU. The simulation numerics and event identification are identical to those described above, with the same time step of $\Delta t = 0.01\,\mathrm{ms}$. To ensure accurate final statistics (in particular BF) each simulation was run for a simulation time of $t_{\max} = 50\,\mathrm{s}$ and the first two seconds were ignored.

As expected, not all parameter choices lead to events. To deal with this, we automatically classified simulation output into one of four basic types: depolarised, hyperpolarised, noisy steady state and event-containing. This was based purely on the dynamics of V. In particular, each simulation was classified as either:

1. Depolarised: range of $V < 10\,\mathrm{mV}$ and mid-range of $V > -50\,\mathrm{mV}$
2. Hyperpolarised: range of $V < 10\,\mathrm{mV}$ and mid-range of $V \leq -50\,\mathrm{mV}$
3. Noisy steady state: range of $V \geq 10\,\mathrm{mV}$ and either (i) no events, (ii) range of $V < 35\,\mathrm{mV}$, or (iii) both the average event gap $< 100\,\mathrm{s}$ and the average event duration more than 2.5 times longer than the average event gap
4. Event-containing: all other cases

In turn, the event-containing cases were classified based on the value of BF as either pure spiking if BF=0, almost pure spiking if 0<BF<0.05, almost pure bursting if 0.95<BF<1, pure bursting if BF=1, or mixed otherwise.

We first tested our result that BF increases with N_{BK} and typically saturates before reaching pure bursting (Fig. 2). This involved picking a random set of parameters as above and running eight simulations with N_{BK} ={1, 2, 3, 4, 5, 9, 20, 45} and $g_{\mathrm{BK}} = g_{1,\mathrm{BK}} N_{\mathrm{BK}}$. Sets were discarded unless (i) each of the eight cases had at least 10 events, (ii) the range of BF was at least 0.5, and (iii) the average burst duration for each case was at least 50 ms. Of the remaining cases, the test was considered passed if BF increased with every increasing value of N_{BK}. A tolerance of 0.1 was permitted since the estimate for BF was based on only $t_{\max} = 50\,\mathrm{s}$ of data. Further, once the maximum BF was reached, the BF for larger values of N_{BK} was allowed to decrease slightly (by up to 0.2 from the maximum value), corresponding to the behaviour seen in Fig. 2.

Next we examined whether BF always increases with cell size (*i.e.* with increasing λ). For each set of parameters, we tested two BK channel densities. In the first, corresponding to approximately 0.0127 BK channels per square-micron of membrane, we compared the cases (a) $\lambda = 0.5, N_{\mathrm{BK}} = 1$,

(b) $\lambda = 1.0, N_{\text{BK}} = 4$ and (c) $\lambda = 1.5, N_{\text{BK}} = 9$. The second test, corresponding to a larger channel density of about 0.0637 channels per square-micron, compared (a) $\lambda = 0.5, N_{\text{BK}} = 5$, (b) $\lambda = 1.0, N_{\text{BK}} = 20$ and (c) $\lambda = 1.5, N_{\text{BK}} = 45$. As above, for each simulation, we set $g_{\text{BK}} = g_{1,\text{BK}} N_{\text{BK}}$. Parameter sets were then rejected unless all simulations had at least 10 events and the average burst duration was at least 50 ms. Finally, each set of three simulations was tested for whether BF increased with λ. As with the first test, we allowed a tolerance of 0.1.

For both our tests, we also considered a significantly wider parameter range than $\pm 50\%$ with $2.5\,\text{pS} \leq g_{1,\text{Ca}} \leq 25\,\text{pS}$, $0\,\text{pS} \leq g_{1,\text{K}} \leq 23\,\text{pS}$, $2.5\,\text{pS} \leq g_{1,\text{SK}} \leq 30\,\text{pS}$, $0\,\text{pS} \leq g_{1,\text{BK}} \leq 400\,\text{pS}$, $0\,\text{nS} \leq g_l \leq 0.5\,\text{nS}$, $-85\,\text{mV} \leq V_l \leq +10\,\text{mV}$ and $0.07\,\text{ms}^{-1} \leq \text{k}_\text{c} \leq 0.35\,\text{ms}^{-1}$. As expected, fewer cases then contained events (although still around 70%), but still around 92% passed the first test and over 80% the second.

7 Phase plots

This section shows some phase plots of the deterministic model. In particular, we plot (i) the membrane potential V against the potassium gating variable n, and (ii) the time-derivative of the membrane potential $\frac{dV}{dt}$ against V. Other plots (involving, for example, m, s, f, [Ca] or I_{X}) are of course possible but are not as informative.

7.1 Changing the cell size

Fig. A shows phase plots for five different cell sizes corresponding to $\lambda = 1.3, 1.366, 1.367, 1.3779$ and 1.539. These should be examined in conjunction with Fig. 5A in the main text. As explained above, the maximal BK conductance scales with λ as $g_{\text{BK}} \to \lambda^2 g_{\text{BK}}$. Although not shown, we set (as usual) $g_{\text{BK}} = 0.5\,\text{nS}$ in the case $\lambda = 1$, which corresponds to pure spiking in the deterministic model. As the cell size increases, there is a transition to bursting at some point between $\lambda = 1.366$ and $\lambda = 1.367$, leading to oscillations around the depolarised state. Further increasing the cell size, leads to extra oscillations within the depolarised state.

7.2 The effect of perturbations

As detailed above, perturbations in the deterministic model are implemented by injecting a current for a short period of time. In Fig. B we consider a perturbation that corresponds to closing seven calcium channels. We consider four different cases: (i) no applied perturbation, (ii) a perturbation applied when V first rises above $-12\,\text{mV}$, (iii) a perturbation applied when V first rises above $-7\,\text{mV}$, and (iv) a perturbation applied when V first drops back

below $-11\,\text{mV}$ (after having first reached its maximum V_{max}). The non-perturbed case corresponds to pure spiking. Many perturbations (such as cases (ii) and (iv)) affect the trajectories but still lead to spiking. However, perturbations within the correct window (such as case (iii); see Fig. 7A in the main text) cause a transition to bursting behaviour. It is worth noting that these perturbations lead to a discontinuity in $\frac{dV}{dt}$ at the points when the perturbation is suddenly applied and then when it is suddenly removed; although the potential V remains continuous, it is no longer differentiable at these points.

Supporting Text References

[1] Tabak J, Tomaiuolo M, Gonzalez-Iglesias AE, Milescu LS, Bertram R (2011) Fast-activating voltage- and calcium-dependent potassium (BK) conductance promotes bursting in pituitary cells: a dynamic clamp study. *J Neurosci* 31(46):16855–16863.

[2] Anderson DF, Ermentrout B, Thomas PJ (2015) Stochastic representations of ion channel kinetics and exact stochastic simulation of neuronal dynamics. *Comput Neurosci* 38:67–82.

[3] Fall CP, Marland ES, Wagner JM, Tyson JJ (2002) Chapter 11 "Modeling the Stochastic Gating of Ion Channels" in *Computational Cell Biology*, Springer-Verlag New York, ISBN 978-0-387-95369-4.

[4] Hille B (2001) Chapters 4 and 5 in *Ion Channels of Excitable Membranes*, Oxford University Press, ISBN 978-0-87893-321-1.

[5] Lee US, Cui J (2010) BK channel activation: structural and functional insights. *Trends Neurosci* 33(9):415–423.

[6] Tagliavini A, Tabak J, Bertram R, Pedersen MG (2016) Is bursting more effective than spiking in evoking pituitary hormone secretion? A spatiotemporal simulation study of calcium and granule dynamics. *Am J Physiol Endocrinol Metab* 310(7):E515–525.

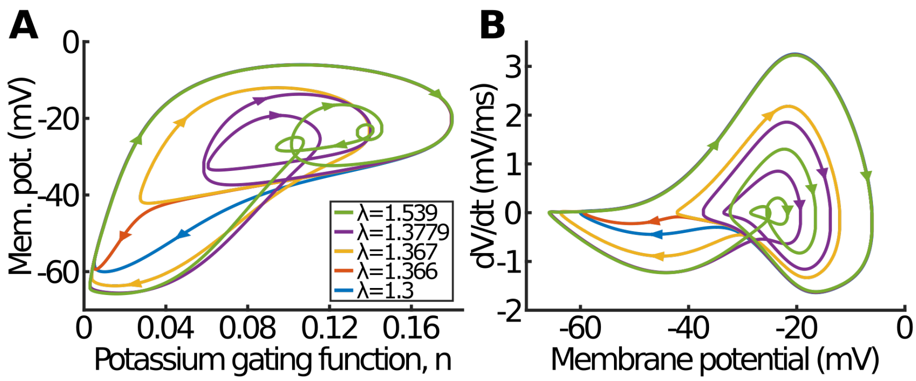

Figure A: **Phase plots in the deterministic model for various cell sizes.** (A) The membrane potential V against the potassium gating variable n for five different cell sizes. The maximal BK conductance is set as $g_{\mathrm{BK}} = 0.5\,\mathrm{nS}$ in the case $\lambda = 1$ (not shown). $\lambda = 1.3$ (like $\lambda = 1$) corresponds to the case of pure spiking. As the cell size increases there is a transition to bursting somewhere between $\lambda = 1.366$ and $\lambda = 1.367$. The oscillation around the depolarised state is clearly seen in the case $\lambda = 1.367$. As the cell size continues to increase, further oscillations occur around the depolarised state. (B) As in (A) but for the time-derivative of the membrane potential $\frac{dV}{dt}$ against V.

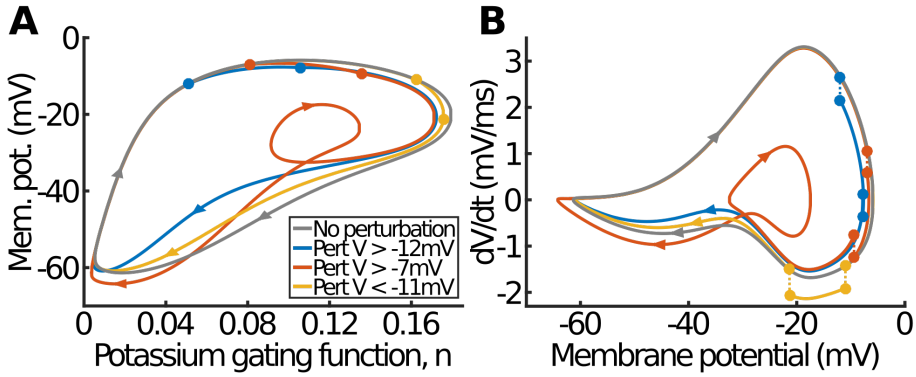

Figure B: **Phase plots in the deterministic model with various applied perturbations.** Here the perturbation in all cases corresponds to closing 7 calcium channels. (A) The membrane potential V against the potassium gating variable n for no perturbation (grey line) and perturbations applied at three different points (coloured lines). Blue: perturbation applied when V first rises above $-12\,\mathrm{mV}$. Red: perturbation applied when V first rises above $-7\,\mathrm{mV}$. Yellow: perturbation applied when V first drops back below $-11\,\mathrm{mV}$ (after first having passed V_{max}). Most perturbations (such as the blue and yellow cases) fail to disturb the spiking behaviour of the non-perturbed case. However, a perturbation applied within the correct window (such as the red curve) can cause spiking to transition into bursting. In each case $g_{\mathrm{BK}} = 0.5\,\mathrm{nS}$. The filled circles represent the points when the perturbation is first applied and the points when the perturbation is removed. (B) As in (A) but for the time-derivative of the membrane potential $\frac{dV}{dt}$ against V. The dashed lines indicate discontinuities in $\frac{dV}{dt}$ at the point the perturbation is first applied or removed.
